# Chirality provides a direct fitness advantage and facilitates intermixing in cellular aggregates

**DOI:** 10.1371/journal.pcbi.1006645

**Published:** 2018-12-27

**Authors:** Ashish B. George, Kirill S. Korolev

**Affiliations:** 1 Department of Physics, Boston University, Boston, Massachusetts, United States of America; 2 Department of Physics and Graduate Program in Bioinformatics, Boston University, Boston, Massachusetts, United States of America; Rice University, UNITED STATES

## Abstract

Chirality in shape and motility can evolve rapidly in microbes and cancer cells. To determine how chirality affects cell fitness, we developed a model of chiral growth in compact aggregates such as microbial colonies and solid tumors. Our model recapitulates previous experimental findings and shows that mutant cells can invade by increasing their chirality or switching their handedness. The invasion results either in a takeover or stable coexistence between the mutant and the ancestor depending on their relative chirality. For large chiralities, the coexistence is accompanied by strong intermixing between the cells, while spatial segregation occurs otherwise. We show that the competition within the aggregate is mediated by bulges in regions where the cells with different chiralities meet. The two-way coupling between aggregate shape and natural selection is described by the chiral Kardar-Parisi-Zhang equation coupled to the Burgers’ equation with multiplicative noise. We solve for the key features of this theory to explain the origin of selection on chirality. Overall, our work suggests that chirality could be an important ecological trait that mediates competition, invasion, and spatial structure in cellular populations.

## Author summary

Is it better to be left- or right-handed? The answer depends on whether the goal is making a handshake or winning a boxing match. The need for coordination favors the handedness of the majority, but being different could also provide an advantage. The same rules could apply to microbial colonies and cancer tumors. Like humans, cells often have handedness (chirality) that reflects the lack of mirror symmetry in their shapes or movement patterns. We find that cells gain a substantial fitness advantage by either increasing the magnitude of their chirality or switching to the opposite handedness. Selection for specific chirality can overcome differences in growth rate and is mediated by the formation of bulges along the colony edge in regions where cells with different chiralities meet.

## Introduction

Living systems have harnessed a variety of physical principles to design and exploit spatial patterns [1–3]. Many biological patterns are chiral, i.e. they break left-right symmetry. While the mechanism of chiral symmetry breaking have been elucidated in some systems [4–10], the functional role of chirality remains largely unexplored [10–13].

Chirality exists at all scales: from molecules to populations [7, 13–18]. The origin of molecular chirality is typically attributed to chance [4, 5, 19, 20]. For nucleotides and amino acids, the classical explanation of homochirality posits two steps: a fluctuation that slightly breaks the left-right symmetry and a self-amplifying process that increases the asymmetry further [6, 19]. More recent work demonstrates that the amplification step may not be necessary because intrinsic noise in chemical reactions is sufficient to establish and stabilize the symmetry breaking [20]. The existence of many chiral components within the cell then serves as a natural explanation for macroscopic chirality [15, 21, 22]. Consistent with this view, chiral body plans arise early in the development due to a symmetry breaking event at a microscopic scale, which is amplified further during the subsequent growth [8, 9, 16, 21, 23, 24]. Similarly, the macroscopic chirality of bacterial colonies is typically explained by the chirality of individual bacteria [13, 25, 26].

The existing theory explains how, but not why chirality emerges. Indeed, a lot of effort went into elucidating the mechanism of the chiral symmetry breaking [7, 13, 14, 27], but the relationship between chirality and fitness has received much less attention [11, 12, 28–30]. Several lines of evidence, however, do suggest that a change in chirality could be advantageous [11, 12, 28–30]. Experiments with *Arthrospira* showed that this bacterium changes from a right-handed to a left-handed helix following the exposure to grazing by a ciliate [11, 28]. Extensive work with *Paenibacillus* demonstrated that this microbe switches between a chiral and a non-chiral forms to optimize its fitness in different environments [13, 30]. Human cells are also known to form chiral patterns. The handedness of these patterns is the same across all tissue types; except, it is reversed in cancer [29]. Thus, in a variety of systems, a change in chirality co-occurs with the evolution of higher growth, dispersal, or competitive ability.

Motivated by these striking examples, we decided to explore whether chirality could be a product of natural selection rather than a historical accident. We asked this question in the context of growing cellular aggregates and found that chirality could directly affect fitness through a pattern-formation mechanism. Our results show that, depending on the growth conditions, it could be advantageous for a cell to increase its chirality or to switch its handedness relative to that of the ancestral population. These dynamics often lead to the coexistence of the left- and right-handed forms, which is a major departure from the classic theories of homochirality [6, 19, 20]. Coexisting cell types may enjoy additional benefits of chirality because they develop unique spatial structure that facilitates cross-feeding and other social interactions [31–34].

## Results

### Model of chiral growth in compact aggregates

Microbial colonies and cancer tumors exhibit a variety of complex morphologies including smooth and rough compact disks, concentric rings, radiating branches, and many others [13, 36–39]. For aggregates that grow as a network of filaments or branches, chirality manifests as clockwise or counterclockwise bending of the branches [27]. Although chiral growth is not as easily detected for other morphologies, it could be present even if the overall colony shape shows no left-right asymmetry.

**Fig 1:**
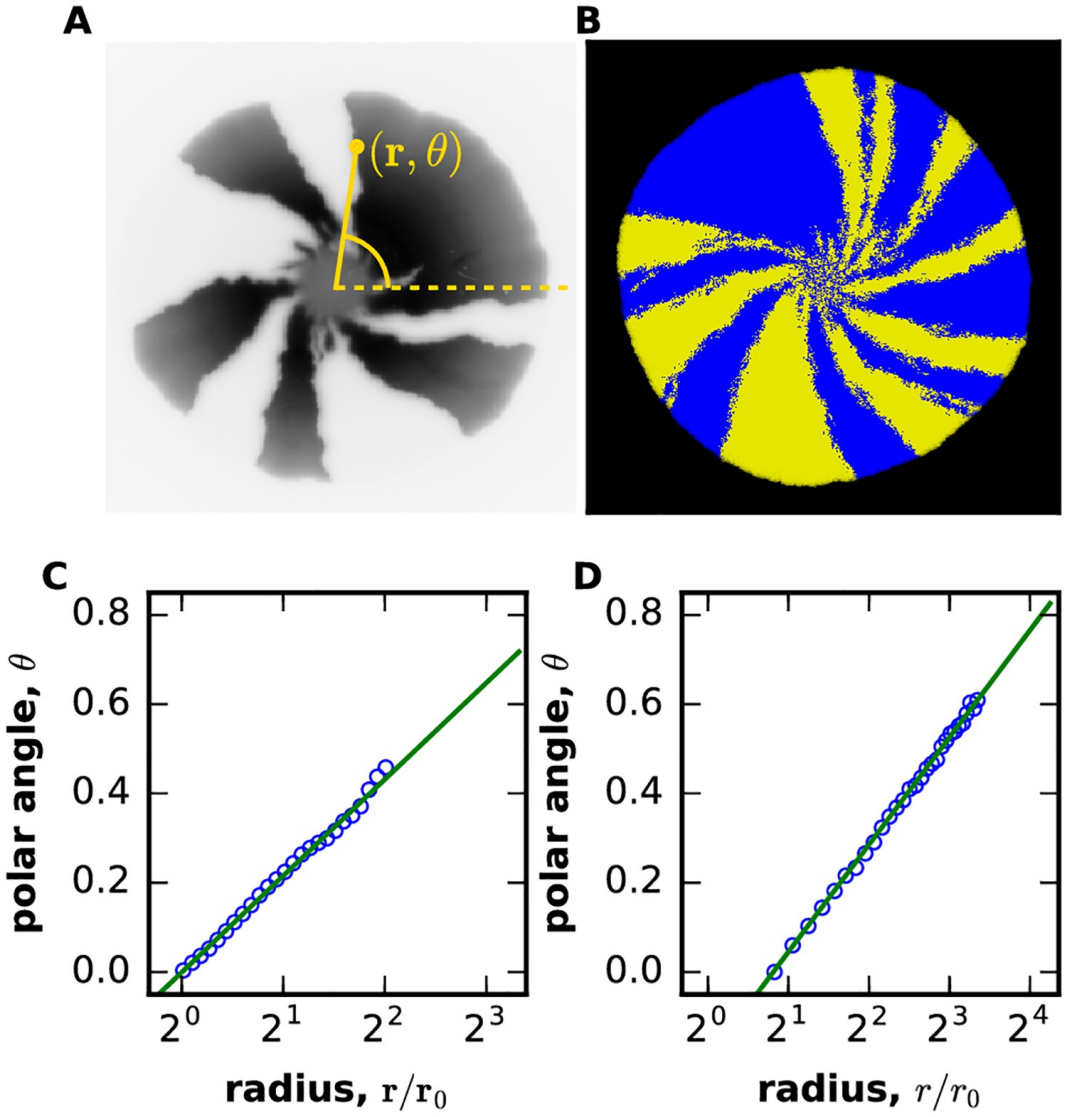
Reaction-diffusion model of chiral growth accurately describes the behavior of sector boundaries in compact microbial colonies. Population dynamics are visualized by the spatial pattern produced during the growth of two neutral strains expressing different fluorescent proteins. The growth is largely limited to the colony edge, so the patterns behind the front do not change over time. Although initially the strains are well-mixed, strong genetic drift leads to local extinctions of one of the strains, which manifests as a characteristic pattern of sectors in both experiments **(A)** and simulations **(B)**. The boundaries between the sectors fluctuate due to genetic drift and twist counterclockwise due to a chiral bias in cell motion. This bias is quantified in **(C)** for experiments and in **(D)** for simulations by plotting the polar angle *θ*, averaged over many sector boundaries, *vs*. the radius *r*. A constant boundary velocity along the colony edge should result in a linear increase of *θ* with ln*r* [35]. Consistent with this expectation, both plots show that sector boundaries are logarithmic spirals. The excellent agreement between experiments and simulations indicates that our reaction-diffusion model is suitable for the study of competition between chiral strains in compact microbial colonies. The experimental data was obtained from the Dryad digital data repository associated with Ref. [18]. Here, *m*_0_ = *m*_*s*_ = *m*_*b*_ = *m*_*d*_ = 0, *g* = 0.03, *N* = 100, *m*_*l*_ = 0.045, *m*_*r*_ = 0.005 for both strains. Radius of initial circle was 30 on a lattice of 700x700 sites.

The “hidden” chirality can be revealed by growing a colony from an initially well-mixed population of two strains that are identical, except they express different fluorescent proteins. As the colony expands, demographic fluctuations at the colony edge lead to local extinctions of one of the strains creating a characteristic pattern of sectors shown in Fig 1A [17, 18, 35, 40, 41]. In the absence of chirality, the boundaries between the sectors are approximately radial, but the boundaries twist consistently clockwise or counterclockwise for chiral cells. The direction of this twisting stays the same across replicate colonies [17, 18, 26]. So far, this method has been applied only to a few model organisms; two of them, *Escherichia coli* and *Bacillus subtilis*, were found to grow in a chiral fashion [17, 18, 26]. Since most organisms have not been examined, hidden chirality could potentially be quite prevalent in cellular aggregates.

The twisting of the boundaries can be quantified by the increase of the polar angle with the distance from the colony center. Using the data from Ref. [18], Fig 1C shows that this dependence is logarithmic, i.e. the boundaries twist as Bernoulli spirals [42]. The origin of the logarithmic twisting can be explained by a simple phenomenological description that combines a constant velocity of the sector boundary with a linear increase of the colony radius in time [35]. The molecular mechanism responsible for cellular chirality has not been fully determined, but we do know that chirality in not due to flagella and is mediated by outer membrane proteins such as antigen 43, extracellular structures including pili, and the interaction with the substratum [26].

Because the factors that mediate chirality also contribute to other components of cell phenotype, it could be challenging to create two strains that differ only in their chirality. This difficulty, however, can be easily overcome in a computational model, where chirality can be tuned without affecting the growth and motility (see Methods and SI). A large number of approaches has been developed to model cellular aggregates from analytic equations to mechanistic simulations [43–47]. We chose to study a minimal reaction-diffusion model because it involves few parameters and is more likely to capture the universal behavior that generalizes across diverse cellular populations. We also focused on the simplest morphology of a compact disk because it is both common and well-understood.

For strains with equal chirality, our simulations (described below) showed excellent agreement with the experimental observations (Fig 1). The simulations not only reproduced the formation and bending of sectors, but also exhibited the same logarithmic twisting of sector boundaries as in the experiments. Thus, the few ingredients in our model are sufficient to describe the chiral growth in compact microbial colonies.

In simulations, cells grow and move in a two-dimensional habitat. The movement is stochastic and short-ranged, but potentially biased relative to the direction of the local density gradient. For non-chiral populations, the bias is along the gradient, in the direction of the outward growth. This bias accounts for the effects of chemotaxis towards nutrients and higher pressure within the colony. For chiral cells, the direction of movement is not collinear with the applied force [48], so the bias in cell movement makes a nontrivial angle with the local gradient of the population density. The sign and magnitude of this angle control the handedness and the strength of chirality. A detailed description of the simulation procedure is provided in Methods and SI.

In the deterministic limit, our simulations are described by the following reaction-diffusion equation:

$$\begin{array}{c} \frac{\partial n^{\left(\alpha \right)}}{\partial t}=g^{\left(\alpha \right)}n^{\left(\alpha \right)}-\nabla \cdot J^{\left(\alpha \right)}, \end{array}$$ (1)

where *t* is time, *n*^(*α*)^ is the population density of strain *α*, *g*^(*α*)^ is a density-dependent per capita growth rate, and the flux *J* is given by

$$\begin{array}{c} J_{i}^{\left(\alpha \right)}=-D^{\left(\alpha \right)}\nabla_{i}n^{\left(\alpha \right)}-n^{\left(\alpha \right)}\sum_{j}(S^{\left(\alpha \right)}\delta_{ij}-A^{\left(\alpha \right)}\epsilon_{ij})\nabla_{j}n. \end{array}$$ (2)

Here, the indexes denote the Cartesian components of vectors, *δ*_*ij*_ is the unit tensor, and *ϵ*_*ij*_ is the totally antisymmetric tensor, also known as Levi-Civita symbol.

The density-dependent diffusion and advection are described by *D*^(*α*)^(*n*) and *S*^(*α*)^(*n*) respectively. *A*^(*α*)^(*n*) is the strength of the chiral term, which is the only term that changes sign under the mirror symmetry. To the lowest order in the gradient expansion, no other term that breaks the left-right symmetry can be added to Eq (2), which suggests that all chiral patterns in compact aggregates are described by our coarse-grained theory regardless of the microscopic origin of chirality.

### Competition between cells with different chirality

**Fig 2:**
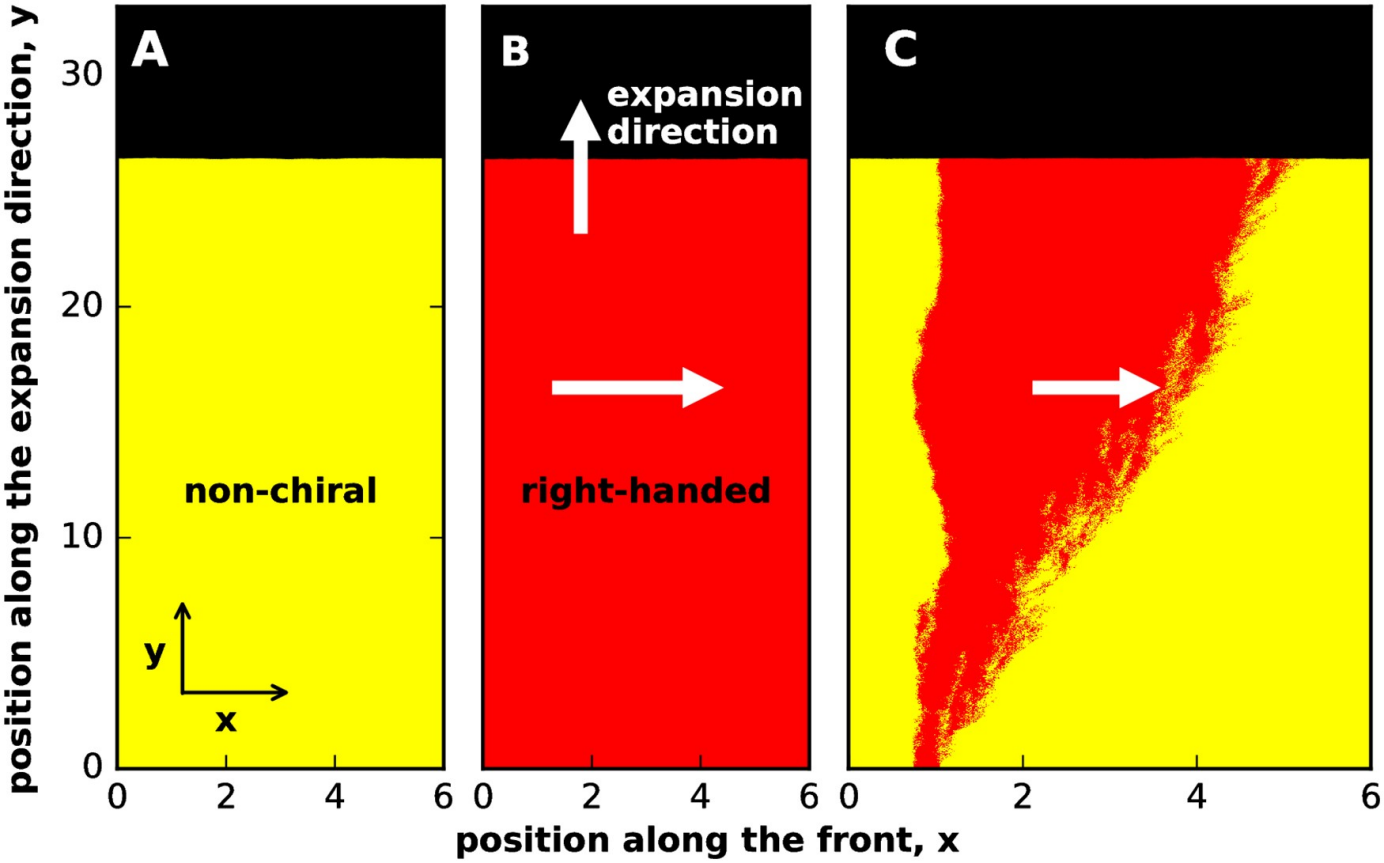
Chirality provides a fitness advantage in competition, but not in overall growth. The first two panels show that a non-chiral **(A)** and a chiral strain **(B)** expand with the same velocity. To facilitate this comparison, we started the simulations with a linear instead of a circular front. Panel **(C)** demonstrates the chiral strain displaces the non-chiral strain when they compete within the same colony: A small initial population of the right-handed strain (shown in red) expands over time and eventually takes over. Because the simulations respect the mirror symmetry, the results were the same irrespective of whether the chirality was due to a left-handed or a right-handed bias in motility. Note that the fate of a strain is not determined solely by its expansion velocity because the colonies expand as pushed waves [47, 49–51]. The expansion velocity of a pushed wave depends not only on the growth and migration rates at the leading edge, but also on the non-linear population dynamics within the wave front. As a result, the outcome of the competition is affected by how each of the strains responds to the presence of the other strain. In our simulations, expansions are pushed because of the density-dependent motility; see Methods and SI. Here, *m*_0_ = *m*_*s*_ = *m*_*b*_ = *m*_*d*_ = 0, *g* = 0.1, *N* = 200 for both strains. $m_{l}^{\left(1\right)}=m_{r}^{\left(1\right)}=0.05$, $m_{l}^{\left(2\right)}=0.01$, $m_{r}^{\left(2\right)}=0.09$ on a lattice of 600x3300 sites. All distances were measured in 100 units where Δx = Δy = 1 unit.

To test whether chirality confers a selective advantage, we competed a chiral strain *vs*. a non-chiral strain. The growth and motility rates of these strains were identical and, as a result, they expanded at the same rate when grown separately (Fig 2A and 2B); see SI for a more quantitative comparison of the velocities. The chiral strain, however, had a clear selective advantage when the competition occurred within the same colony. Fig 2C illustrates this by showing how the chiral strain took over the population starting from a small, localized patch representing a mutation or an immigration event. This selective advantage of chirality was not specific to the competition between a chiral *vs*. strictly non-chiral strains. In simulations, we explored a wide range of microscopic parameters and invariable found that the more chiral strain outcompeted the less chiral strain *when both strains had the same handedness*.

**Fig 3:**
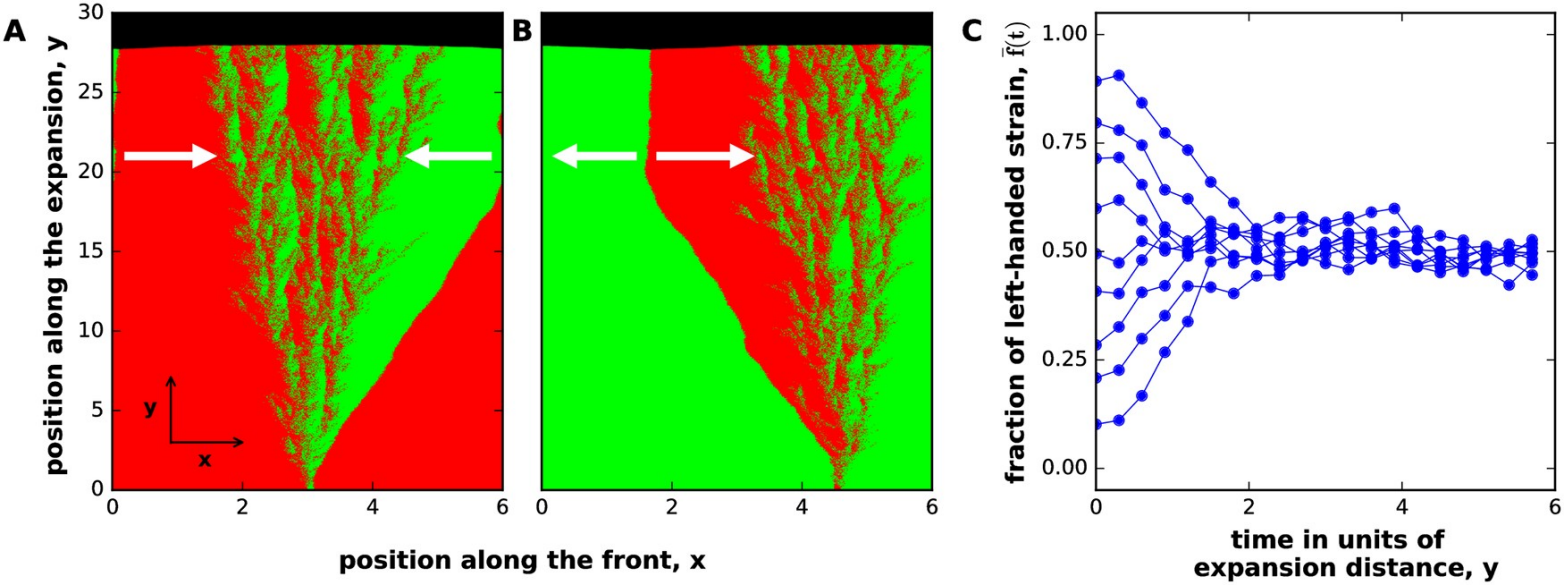
Selection for coexistence between strains with opposite handedness. Panel **(A)** shows that a left-handed mutant (shown in green) can invade a right-handed population (shown in red). The reverse invasion also occurs and is shown in panel **(B)**. This negative frequency-dependent selection is further illustrated in panel **(C)**, which shows how $\bar{f}$, the spatially averaged relative abundance of the first strain, changes over time starting from different initial conditions. At *t* = 0, the strains are spatially separated in (A) and (B), but well-mixed in (C). In this figure, the strains have exactly opposite chiralities, but coexistence occurs more generally; see Fig 7. Note that the selection for coexistence relies on the presence of boundaries between the strains. When strains intermix (as shown in this figure), we observe a strong and time-invariant selection for coexistence. When strains do not intermix (see Figs 5 and 6), the number of boundaries slowly declines over time due to neutral coarsening [18, 41]. In such cases, robust coexistence relies on occasional external re-mixing events, e.g. during the establishment of a new colony. Here, *m*_0_ = *m*_*s*_ = *m*_*b*_ = *m*_*d*_ = 0, *g* = 0.1, *N* = 200 for both strains. $m_{l}^{\left(2\right)}=0.09$, $m_{r}^{\left(2\right)}=0.01$, $m_{l}^{\left(2\right)}=0.01$, $m_{r}^{\left(2\right)}=0.09$ on a lattice of 600x3000 sites. All distances were measured in 100 units where Δx = Δy = 1 unit.

In contrast, the competition between two strains with *opposite handedness* often resulted in stable coexistence. As an example of this, Fig 3 shows the competition between two strains with chiralities that equal in magnitude, but opposite in direction. Both strains invaded when introduced in the population of the opposite handedness, but did not completely take over (Fig 3A and 3B). Instead, the population approached a steady state where both strains were equally abundant. To confirm this observation of negative frequency-dependent selection, we performed simulations starting from well-mixed initial conditions with different fractions of the left-handed strain. As expected from symmetry, the fraction of the left-handed strain converged to 50% (Fig 3C) suggesting that left- and right-handed strains can stably coexist. Strains with opposite, but not exactly equal chirality were also found to coexist, but the equilibrium fractions deviated from the 50:50 ratio in favor of the more chiral strain.

**Fig 4:**
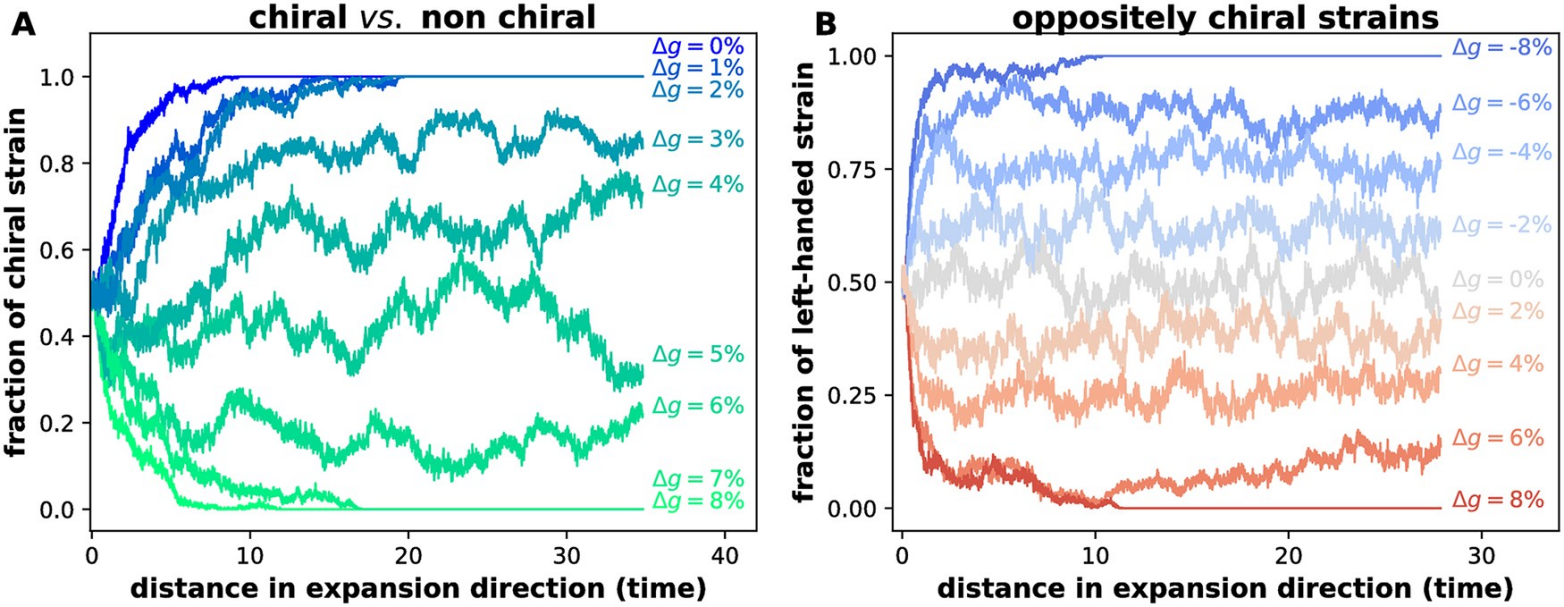
Effects of chirality persist despite growth rate differences. We competed strains with different growth rates and chirality starting from well-mixed initial conditions. The abundances of the strains were equal at the beginning of the simulations, but changed over time leading either to stable coexistence or to the exclusion of one of the strains. The outcome of the competition depends on the relative growth rates of the strains quantified by Δ*g*. The competition between a left-handed strain and a faster growing non-chiral strain is shown in **(A)**. The non-chiral strain is outcompeted even if it has a growth advantage as high as to 2%. The chiral strain becomes extinct only when its growth penalty exceeds 7%. For intermediate Δ*g*, the two strains stably coexist. Similar dynamics occur during the competition between the strains with equal, but opposite chirality shown in panel **(B)**. The stable coexistence between the strains is destroyed only by growth rate differences higher than about 7%. Here, *m*_0_ = *m*_*s*_ = *m*_*b*_ = *m*_*d*_ = 0 for both strains and *N* = 200. In (A), $m_{l}^{\left(1\right)}=0.1$, $m_{r}^{\left(1\right)}=0.0$, $m_{l}^{\left(2\right)}=0.05$, $m_{r}^{\left(2\right)}=0.05$ on a lattice of 1000x3600 sites. We fixed *g* = 0.01 for the left handed-strain and varied the growth rate of the non-chiral strain according to *g*(1 + Δ*g*/100%). In (B), $m_{l}^{\left(1\right)}=0.09$, $m_{r}^{\left(1\right)}=0.01$, $m_{l}^{\left(2\right)}=0.01$, $m_{r}^{\left(2\right)}=0.09$ on a lattice of 500x3000 sites. We set *g* = 0.1 for the left-handed strain, and varied the growth rate of the right-handed strain according to *g*(1 + Δ*g*/100%). The distances on the x-axis were measured in 100 units where Δx = Δy = 1 unit. We verified stable coexistence by starting the simulation above and below the observed steady-state relative abundances and checking that they return to the same steady-state values.

The effects of chirality persist even when the strains have different growth rates. To demonstrate this, we competed a chiral *vs*. a faster growing non-chiral strain (Fig 4A). The chiral strain completely excluded the non-chiral strain provided its growth rate penalty was less than 2%. For growth rate differences between 2% and 7%, the two strains stably coexisted. The chiral strain went extinct only when its growth rate penalty exceeded 7%. Similar dynamics occurred during the competition between two strains with opposite handedness (Fig 4B). As we increased the difference in the growth rate between the strains, their steady-state abundance started to deviate from the 50:50 ratio. The coexistence was lost only when the growth rate difference exceeded 7%. The 7% threshold is representative for the parameters used in our reaction-diffusion model, but its value in an actual biological system could be affected by the details of the cell-cell and cell-surface interactions, which we did not explicitly model. Nevertheless, our results demonstrate that chirality can influence the outcome of the competition between strains with substantial differences in growth and motility.

Selection mediated by chirality may seem quite surprising. In the following, we first explain the origin of this phenomenon using the competition between strains with opposite handedness as a simple example. After that, we return to the general case and explore the transition between exclusion and coexistence as the chirality of the strains is varied. We also describe the role of demographic fluctuations and characterize the spatial patterns that emerge in populations of chiral cells.

### Effective theory of chiral growth

Why does chirality affect competition? To answer this question, we developed an analytical theory that explains the spatial patterns shown in Figs 1–3. For this purpose, we reduced the reaction-diffusion model (Eq (1)) to a simpler effective theory that describes only the overall shape of the colony edge and its genetic composition (see SI). This effective theory can also be derived purely from the symmetry considerations and is therefore more general than the underlying reaction-diffusion model (see SI). Below, we use the effective theory to explain how the competition between two strains is affected by their chiralities. Our main result is that the difference in chiralities leads to changes in colony shape, which in turn influence the relative abundance of the strains.

Because little growth occurs inside cellular aggregates [47, 52], their population dynamics can be largely described in terms of only two variables: the position of expansion front and the relative abundance of the strains at the edge of the colony. To fix the coordinate system, we take the *x*-axis to be a straight line along the average direction of the colony edge (Fig 2A). The *y*-axis then points in the direction of colony growth. We denote the *y*-coordinate of the expansion front by *h*(*t*, *x*), and the fraction of the first strain by *f*(*t*, *x*). In terms of these quantities, the effective theory is given by the following set of equations:

$$\begin{array}{cc} & \frac{\partial h}{\partial t}=v_{0}+\frac{v_{0}}{2}{\left(\frac{\partial h}{\partial x}\right)}^{2}+D_{h}\frac{\partial^{2}h}{\partial x^{2}}+\alpha \frac{\partial f}{\partial x}+\text{noise},\\ & \frac{\partial f}{\partial t}=D_{f}\frac{\partial^{2}f}{\partial x^{2}}+\beta \left(f^{*}-f\right)\frac{\partial f}{\partial x}+v_{0}\frac{\partial h}{\partial x}\frac{\partial f}{\partial x}+\text{noise}. \end{array}$$ (3)

The first equation in Eq (3) is an extension of the Kardar-Parisi-Zhang (KPZ) equation of surface growth [53–55]. Here, the first term, *v*_0_, is the expansion velocity of the strains grown in isolation. The second term accounts for the fact that the expansion of a tilted front ($\frac{\partial h}{\partial x}\neq 0$) occurs perpendicular to the front and, therefore, at a angle with the *y*-axis. The third term arises because fronts that are curved outward expand more slowly and because of effective surface tension at the edge of the microbial colony [56]. The last term couples the dynamics of *f* and *h* and is a new term that describes chirality. Its magnitude is controlled by parameter *α*, which is proportional to the difference in the chiralities of the strains. We show below that this last term changes colony shape and mediates the competition between chiral strains.

The second equation in Eq (3) is an extension of the Burgers’ equation used to describe fluid and traffic flow [57–60]. The first term describes random, diffusion-like movement, while the second term accounts for the directional motion due to a chiral bias in motility. Here, the factor of −*β*(f* − *f*) can be viewed as a local advection velocity, which depends on the relative abundance of the strains and two parameters that describe the chiral properties of the strains. The first parameter, *β*, is proportional to the difference in the chiralities of the strains. The second parameter f* is the ratio of the chirality of the first strain to the difference in the chiralities of the strains.

We choose the most left-handed (also the least right-handed) strain to be the first strain, which ensures that *α* and *β* are positive (see SI). With this convention, the first strain that is left-handed for f* > 0 and right-handed for f* < 0. When f* ∈ (0, 1), the two strains have opposite handedness, while, when f* ∈ (−∞, 0) ∪ (1, + ∞), the two strains have the same handedness. Furthermore, f* = 0 corresponds to a non-chiral and a right-handed strain, and f* = 1 corresponds to a left-handed and a non-chiral strain. The special case of equal, but opposite chiralities corresponds to f* = 1/2.

The third term in the equation for $\frac{\partial f}{\partial t}$ describes how colony shape affects the relative abundance of the strains. This term is non-zero only in tilted regions of the front, where cells at higher *h* displace cells at lower *h* as the colony grows. In other words, the relative abundance of the strains changes because the growth of the colony proceeds in the direction perpendicular to the front and, therefore, induces the movement of cells along the *x*-axis whenever $\frac{\partial h}{\partial x}\neq 0$. Below we demonstrate how this coupling between colony shape *h*(*x*) and genetic composition *f*(*x*) mediates the competition between the strains in compact aggregates.

Finally, the noise terms account for demographic fluctuations and genetic drift. In the first equation, the noise is the regular additive noise present in the KPZ equation; it arises due to local fluctuations in the growth velocity. In the second equation, the noise accounts for genetic drift, so it is multiplicative with the strength proportional to $\sqrt{f(1-f)}$. Such dependence on *f* is typical for population dynamics [41] and is necessary to ensure that *f* = 0 and *f* = 1 are absorbing states.

### Bulges and dips at sector boundaries

Genetic drift in the equation for *f* leads to local extinctions of one of the strains and the formation of sector boundaries [41]; see Fig 1. When these boundaries separate strains with different chirality, *h*(*t*, *x*) develops this characteristic shape that ultimately controls the competition between the strains. For simplicity, we first discuss the behavior of two strains with exactly opposite chiralities (f* = 1/2). In this special case, the mirror symmetry ensures that the boundaries between the strains do not have a net bias and, therefore, remain stationary when $\frac{\partial h}{\partial x}=0$.

**Fig 5:**
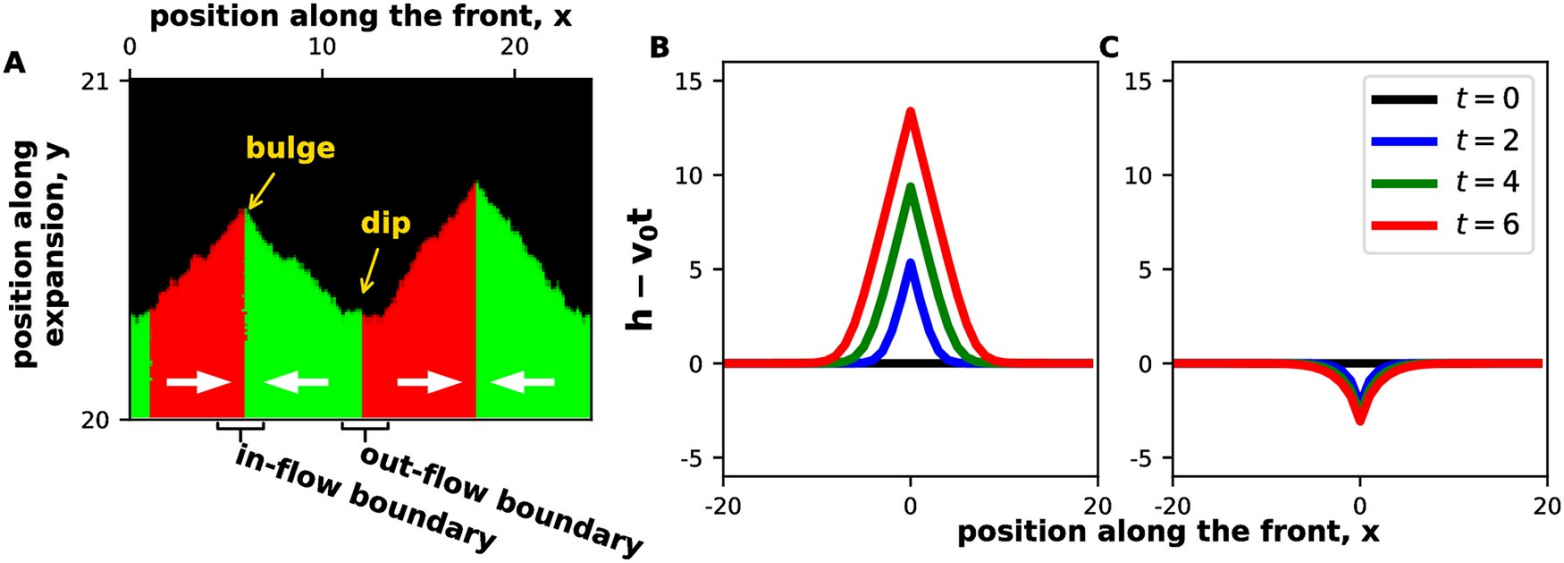
Boundaries between strains with different chiralities create front undulations. **(A)** A magnified view of the colony front shows bulges and dips near in-flow and out-flow boundaries. The analytical solutions for the shape of bulges and dips are shown in panels **(B)** and **(C)** respectively. Note that both the theory and the simulations predict an approximately triangular bulge shape. Here, *m*_0_ = *m*_*s*_ = *m*_*b*_ = *m*_*d*_ = 0, *g* = 0.1, *N* = 200 for both strains, and $m_{l}^{\left(1\right)}=0.009$, $m_{r}^{\left(1\right)}=0.001$, $m_{l}^{\left(2\right)}=0.001$, $m_{r}^{\left(2\right)}=0.009$ on a lattice of 2400x2100 sites in panel(A). The chirality of the strains is shown with thick white arrows. All distances were measured in 100 units where Δx = Δy = 1 unit.

The dynamics of a strain boundary depends on whether the chiral biases of the strains point towards or away from it. We term a boundary an in-flow boundary when the strains move towards each other, i.e. the left-handed strain is to the right of the boundary, and the right-handed strain is to the left of the boundary (Fig 5). Boundaries with the opposite arrangement of the strains are termed out-flow boundaries.

Eq (3) predicts that the two types of boundaries have a diametrically opposite effect on the colony shape. For in-flow boundaries $\alpha \frac{\partial f}{\partial x}>0$, and we expect a bulge due to local overgrowth of *h*. In contrast, a dip in the front is expected at out-flow boundaries, where $\alpha \frac{\partial f}{\partial x}<0$. Fig 5 shows that these shapes indeed develop in our simulations.

We exactly solved the chiral KPZ equation without noise and obtained an analytical expression for the shapes of the bulges and dips (see SI) in the limit of sharp boundaries between the strains. After a transient, the bulges assume an approximately triangular shape given by

$$\begin{array}{c} h\left(t,x\right)-v_{0}t=\{\begin{array}{cc} & 0,\;\;|x-x_{b}|\geq \frac{v_{0}\alpha }{4D_{h}}t,\\ & \frac{v_{0}\alpha^{2}}{8D_{h}^{2}}t-\frac{\alpha }{2D_{h}}|x-x_{b}|,\;\;|x-x_{b}|<\frac{v_{0}\alpha }{4D_{h}}t \end{array} \end{array}$$ (4)

for an in-flow boundary located at *x*_*b*_. The slope of the bulge stays constant, but its height and width increase linearly in time. The depth of the dips, on the other hand, increases only logarithmically in time, so they remain quite small on the time scale of our simulations (see SI). As a consequence, the front primarily consists of titled regions near the bulges and flat regions away from the strain boundaries (Fig 5).

One immediate consequence of Eq (4) is that a mixture of two strains with opposite handedness expands faster than either strain in isolation. Indeed, $dh(t,x_{b})/dt=v_{0}+v_{0}\alpha^{2}/(8D_{h}^{2})$, which is greater than *v*_0_. In our simulation, this increase was typically on the order of a few percent; see SI for further details.

### Origin of selection

**Fig 6:**
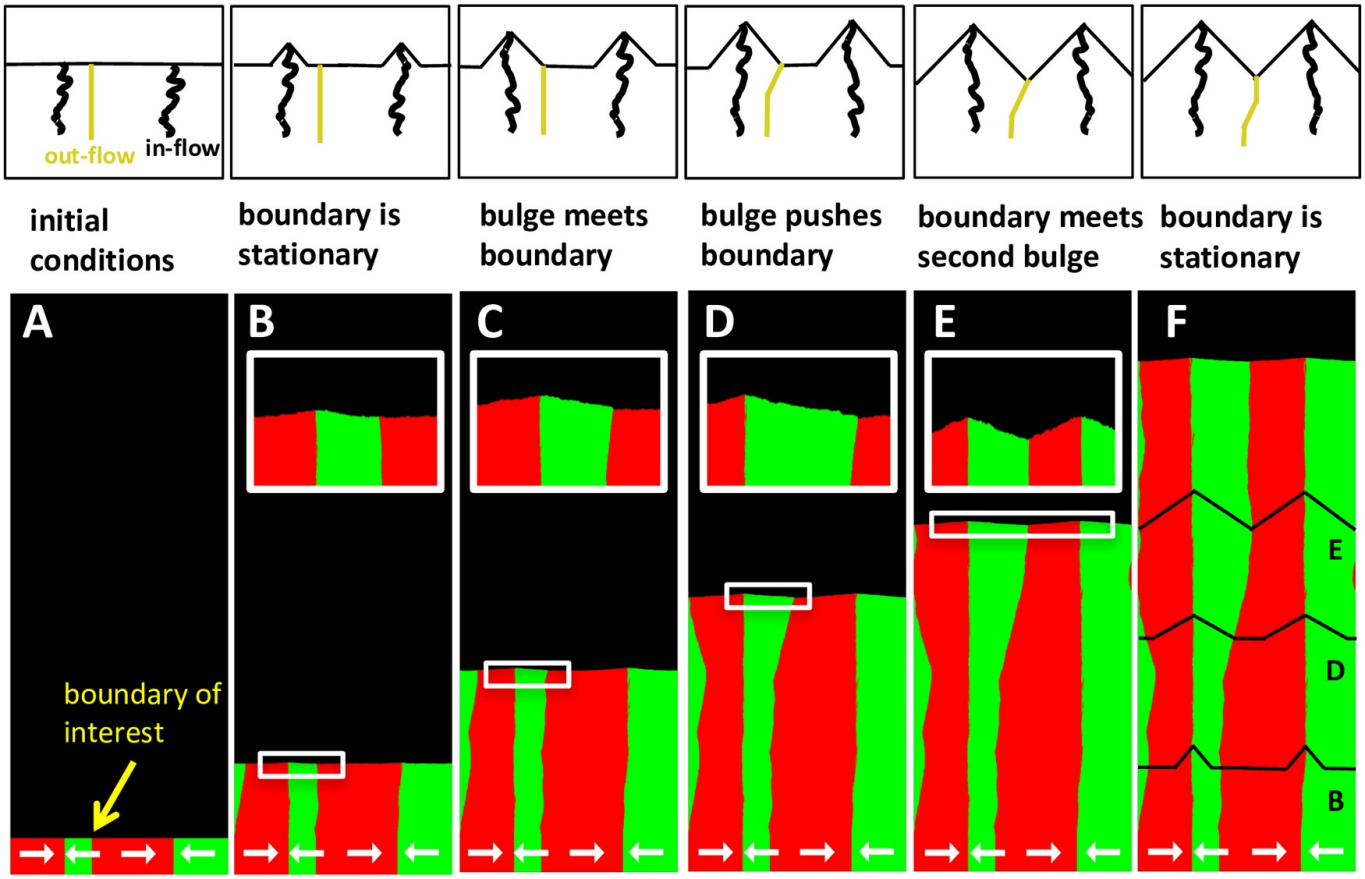
Bulges drive selection by “pushing” out-flow boundaries. The panels show the motion of an out-flow boundary due to the expansion of the bulges formed at the surrounding in-flow boundaries. Initially, the bulges are small **(AB)**. Bulge growth has no effect, until one of the bulges comes in contact with the boundary of interest **(C)**. After that, the expansion of the bulge displaces the out-flow boundary **(D)**. The movement of the boundary stops when it is locked between two nearest bulges **(EF)**. The relationship between the location of bulge and the motion of the out-flow boundary is further clarified in schematics above each of the panels. The black lines in the last panel show the locations (but not the actual size) of the bulges at earlier times. To illustrate the dynamics most clearly, we chose the model parameters in the no-mixing regime (see Fig 8). As a result, both in-flow and out-flow boundaries appear almost equally sharp. The chirality of the strains is shown with thick white arrows. Here, *m*_0_ = *m*_*s*_ = *m*_*b*_ = *m*_*d*_ = 0, *g* = 0.1, *N* = 200 for both strains. $m_{l}^{\left(2\right)}=0.009$, $m_{r}^{\left(2\right)}=0.001$, $m_{l}^{\left(2\right)}=0.001$, $m_{r}^{\left(2\right)}=0.009$ on a lattice of 2400x6400 sites.

The expansion of bulges changes the relative abundance of the strains (Fig 6). Initially, no bulges are present because we start our simulations with a flat front to mimic the coffee-ring effect that creates a smooth edge around a microbial colony [61]. As the colony expands, small bulges form and grow around the in-flow boundaries. In the beginning, a small bulge has no effect on the genetic composition of the front because it is completely enclosed within the two sectors surrounding the in-flow boundary. However, as the bulge expands, it comes in contact with the out-flow boundaries on its sides and then starts “pushing” them outwards. The subsequent movement of the out-flow boundaries changes sector sizes and, therefore, the relative abundance of the strains.

For the case of f* = 1/2 that we are considering now, the left and the right ends of the bulge are equidistant from the in-flow boundary. Hence, the two strains have equal abundance within the bulge. The expansion of the bulge then brings the global fraction of the first strain, $\bar{f}$, towards 1/2. The change in $\bar{f}$ ceases only when the out-flow boundaries stop moving, which occurs when they are locked between the two neighboring bulges (see Fig 6). At this point, the entire front consists of bulges, so $\bar{f}=1/2$.

**Fig 7:**
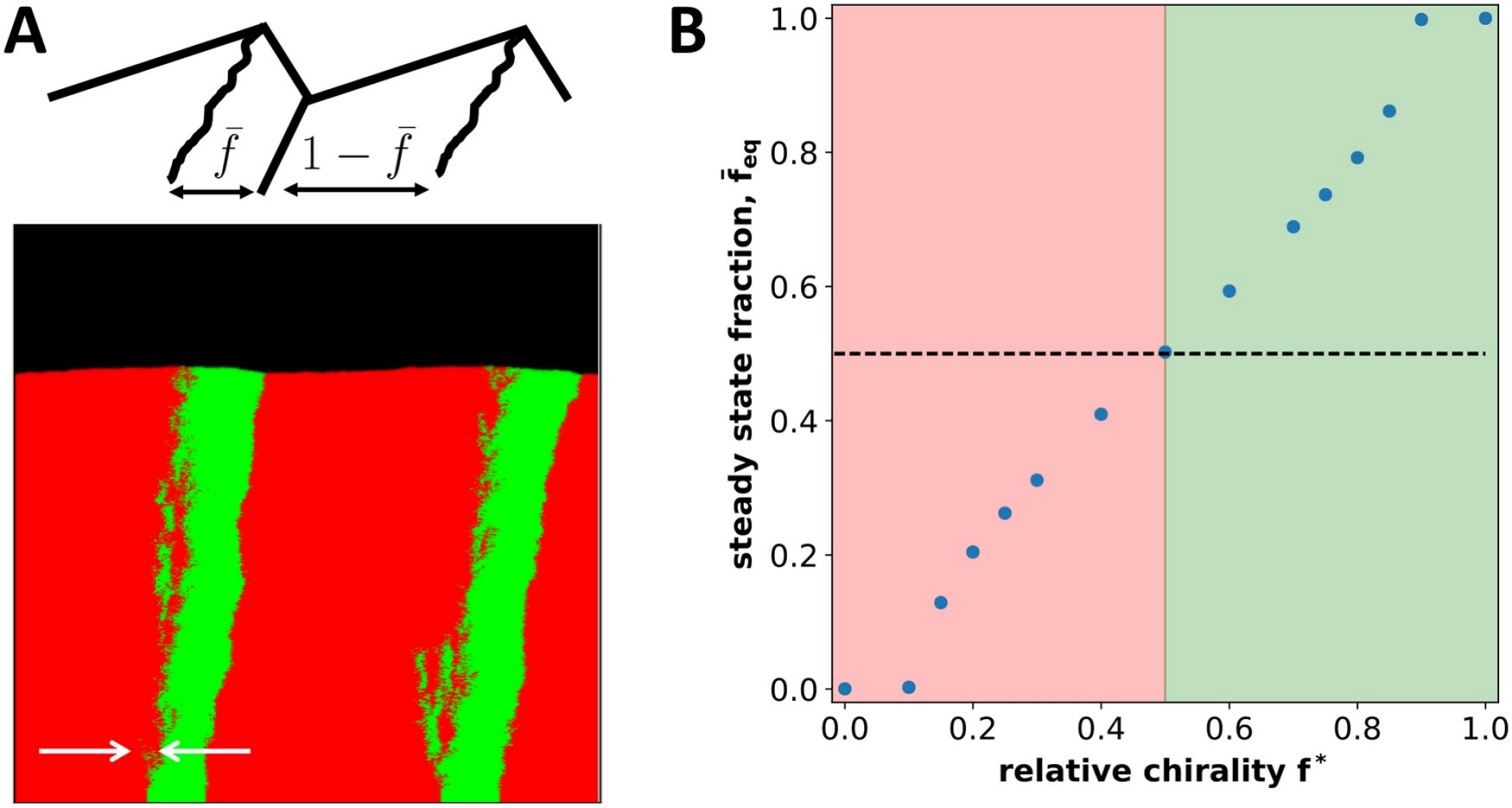
Equilibrium fractions change with relative chirality. **(A)** shows the steady-state spatial structure for two strains with opposite handedness, but unequal magnitudes of chirality (f* ≠ 1/2). **(B)** shows the relative abundance of the strains as a function of their relative chiralities. This relationship is approximately linear in agreement with Eq (5); see also S1 and S2 Figs. Here, *m*_0_ = *m*_*s*_ = *m*_*b*_ = *m*_*d*_ = 0, *g* = 0.1 for both strains. The chirality of the strains is shown with thick white arrows. In (A) $m_{l}^{\left(2\right)}=0.0$, $m_{r}^{\left(2\right)}=0.05$, $m_{l}^{\left(2\right)}=0.00725$, $m_{r}^{\left(2\right)}=0.0425$, *N* = 100. In (B), the chirality of the strains was varied with the difference in their chiralities $(m_{l}^{\left(1\right)}-m_{r}^{\left(1\right)})-(m_{l}^{\left(2\right)}-m_{r}^{\left(2\right)})=0.1$ and *m*_*l*_ + *m*_*r*_ = 0.1 at *N* = 400. Length of lattice was varied to ensure steady state was reached.

The argument above explains the mutual invasion and coexistence for strains with exactly opposite chiralities shown in Fig 3. For f* ≠ 1/2, the dynamics are essentially the same with only two minor modifications (see SI). First, in-flow and out-flow boundaries do not remain stationary within the regions of flat front. Instead, the boundaries move with velocity *v*_∥_ = *β*(1/2 − f*), which reflects the unequal chiral biases of the two strains. Second, while the bulges remain triangular, they are no longer symmetric relative to the *y*-axis. The steeper slope occurs on the side that leads the forward motion of the bulge (Fig 7A).

As before, natural selection occurs due to the expansion of bulges, and the steady state is reached when bulges occupy the entire front. In this state, the relative abundance of the strains is determined by the ratio of bulge slopes, and we find that (see SI)

$$\begin{array}{c} {\bar{f}}_{\text{eq}}=\{\begin{array}{cc} & 0,\;\;f^{*}\leq \frac{1}{2}-\frac{\alpha v_{0}}{2D_{h}\beta },\\ & \frac{1}{2}+\frac{\beta D_{h}}{\alpha v_{0}}(f^{*}-\frac{1}{2}),\;\;|f^{*}-\frac{1}{2}|<\frac{\alpha v_{0}}{2D_{h}\beta }\\ & 1,\;\;f^{*}\geq \frac{1}{2}+\frac{\alpha v_{0}}{2D_{h}\beta }. \end{array} \end{array}$$ (5)

Here, the middle line describes the relative abundance of the strains when they coexist. The first and the last line describe to the exclusion of the less chiral strain. Exclusion occurs when one of bulge slopes becomes horizontal, and, therefore, no steady state can be reached.

These theoretical conclusions are supported by the simulation results summarized in Fig 7B. The data shows a clear transition from coexistence to competitive exclusion and a nearly linear dependence of $\bar{f}$ on f* in the coexistence region as predicted by Eq (5). Quantitative comparison between the theory and the simulations is described in S1 Fig. Close to the extinction transitions, however, there are noticeable deviations from linearity. Such nonlinearities are typical for non-equilibrium phase transitions and are described by critical exponents [31, 62]. To obtain the critical exponent and characterize the nature of the phase transition, one would need to account for the stochastic creation and annihilation of domain boundaries, which we have neglected in our analysis.

The size of the coexistence regions depends on the model parameters (see S2 Fig for a narrower coexistence range). In all of our simulations, we found that the transition between exclusion and coexistence occurs for f* ∈ (0, 1). Therefore, the competition between a chiral and a non-chiral strain falls outside the coexistence region, which explains the competitive exclusion shown in Fig 2.

### Transition to strain intermixing

So far, our analysis has relied on the existence of sharp boundaries between the strains. Such boundaries appear readily due to genetic drift both in microbial colonies [17] and in our simulations (Figs 2 and 5–7). Previously, it has been shown that any non-zero genetic drift prevents diffusive broadening and ensures a finite size of a boundary between two neutral, non-chiral strains [63]. We found that the behavior is unchanged when the two strains have non-zero, but identical chirality (S3 Fig). This observation is not surprising because the front of the colony remains flat, and the chiral motion of the strains can be removed by changing into a reference frame moving along the front. The boundaries also have a finite width (at least on the time scale of our simulations) when one of the strains is outcompeted due to the differences in growth rates or chirality; see S4 Fig and Refs. [35, 64]. In this case, the boundary width is controlled both by genetic drift and selection.

**Fig 8:**
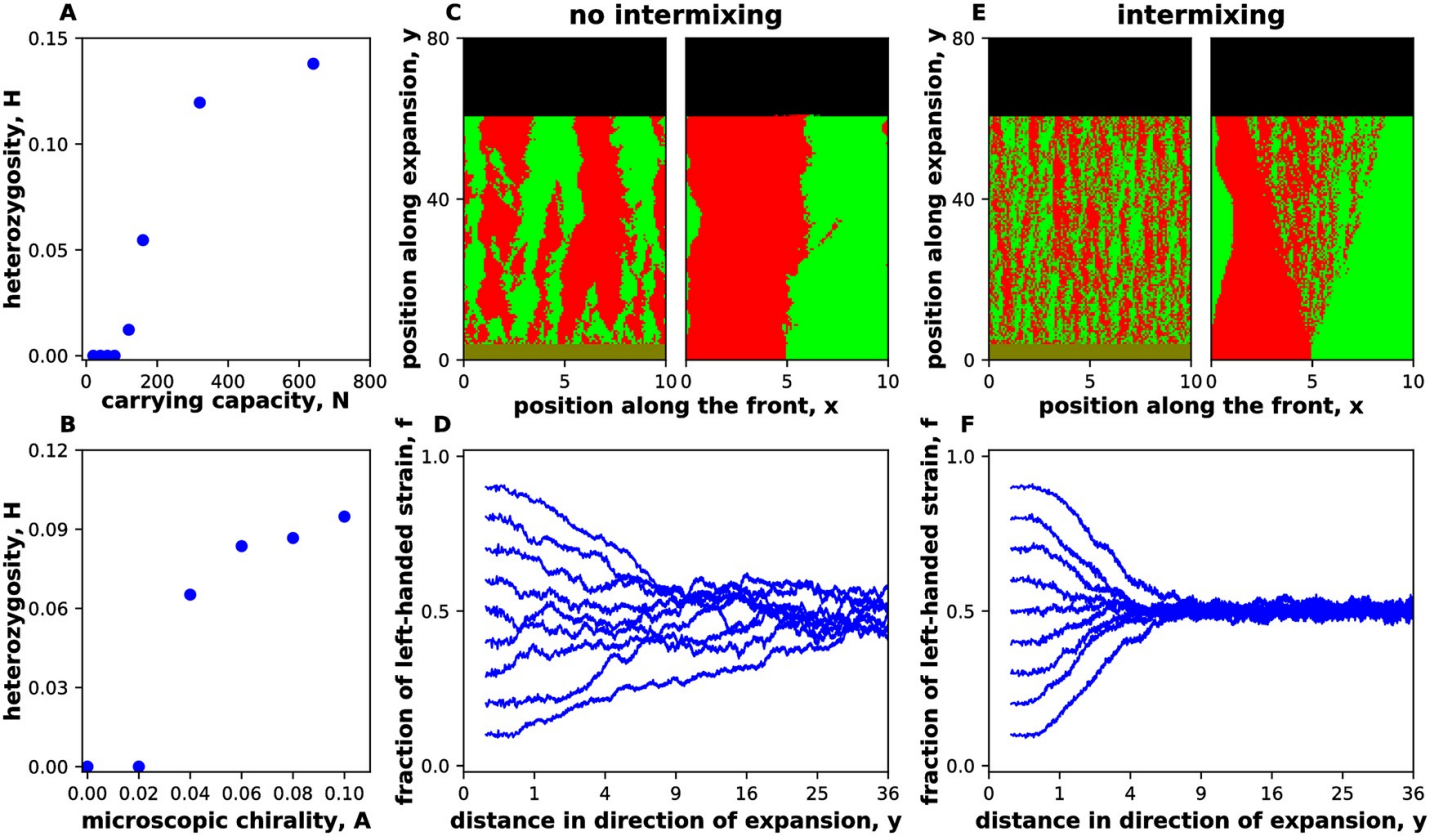
Transition from segregation to strain intermixing. The intermixing of the strains was quantified by heterozygosity *H* = 〈2*f*(1 − *f*)〉, which is nonzero only when both strains are present at the same spatial location. **(A,B)** show that there is a phase transition between an intermixed regime, where *H* has a nonzero value at steady state, and a regime, where the strains spatially segregate with *H* vanishing in the long-time limit. The transition is controlled by the relative magnitude of strain chirality and the strength of genetic drift. The latter depends on the number of the organisms at the growing edge. **(C)** shows the spatial patterns in the demixed regime starting either from a single boundary or from well-mixed initial conditions. **(D)** shows how the relative abundance of the strains change starting from well-mixed initial conditions and different initial fractions. Note that, even in the demixed regime, there is negative frequency-dependent selection towards coexistence. **(E,F)** are the same as (C,D) but in the intermixed regime. All data in this figure are for strains with opposite handedness, but equal magnitude of chirality. Similar results were obtained for unequal magnitudes of chirality; see S4 Fig. Here, *m*_0_ = *m*_*s*_ = *m*_*b*_ = *m*_*d*_ = 0, *g* = 0.1 for both strains. In panels (A), (C), (D), (E), (F), $m_{l}^{\left(2\right)}=0.075$, $m_{r}^{\left(2\right)}=0.025$, $m_{l}^{\left(2\right)}=0.025$, $m_{r}^{\left(2\right)}=0.075$ on a lattice of 1000x8000 sites with *N* = 40 and *N* = 320 for no intermixing and intermixing respectively. In (B) $m_{r}^{\left(1\right)}+m_{l}^{\left(1\right)}=m_{r}^{\left(2\right)}+m_{l}^{\left(2\right)}=0.1$, *N* = 200 was held fixed as chirality was varied. All distances were measured in 100 units where Δx = Δy = 1 unit. The right-handed strain is shown in red, and the left-handed strain is shown in green. Note that the equilibration of *H* requires that *f* is in steady state; therefore, *H* equilibrates more slowly.

When there is selection towards coexistence, there are two distinct possibilities: Either the boundaries are sharp as in Fig 6 or the boundaries widen over time leading to a intermixed state as in Fig 3. We found that there is a well-defined transition between these two regimes, which is controlled by the strength of chirality and genetic drift (Fig 8 and S4 Fig). For strong genetic drift or weak chirality, the boundaries between the strains remain sharp, and population dynamics are completely described by the theory developed above. For weak genetic drift or strong chirality, the strains become intermixed. Our main conclusions remain the same even in this regime (see S5 Fig). In particular, we still observe either coexistence or exclusion depending on the relative chiralities of the strains. The spatial patterns are also similar. A large, non-triangular bulge forms around the intermixed region between the two strains, and small bulges are visible around individual in-flow boundaries (Figs 3 and 8).

The transition between demixed and intermixed regimes appears to be continuous (second order) as seen from Fig 8A. Identification of the universality class of this phase transition and the quantitative description of the mixing regime, however, require a careful analysis of the interplay between stochastic and deterministic terms in Eq (3) and are left for future work.

Spatial intermixing could be especially important for species that participate in social interactions such the exchange of metabolites. In such situations, chirality could not only stabilize the coexistence of the species, but also ensure that they are sufficiently close to each other. When no special mechanism exists to ensure spatial proximity, mutualistic interactions can be easily destroyed by genetic drift [31–34]. Hence, some microbes may rely on different or fluctuating handedness to ensure that the separation between the species does not exceed the maximal distance over which they can interact.

## Discussion

Selection for a particular chirality may seem impossible because an object and its mirror image have identical physical properties. This apparent paradox is however easily resolved by noticing that natural selection always favors a change in chirality relative to that of the ancestral population rather than an absolute, pre-defined value of chirality.

A vivid example of how evolution drives a change in chirality comes from *Satsuma* snails. Most species in the *Satsuma* genus are dextral (clockwise coiled), but they often have sister species that are almost identical except for the opposite direction of coiling [12]. These sinister (counterclockwise coiled) species enjoy a distinct selective advantage because they are essentially resistant to the predation by *Pareatidae iwasakii*, a snake that is common in the rage of *Satsuma* [12]. Resistance to predation comes from the left-right asymmetry in the jaw of *P. iwasakii*, which has adapted to the coiling direction of its most common prey. Similarly, a reversal of handedness provides protection to *Arthrospira*, cyanobacteria that forms helical trichomes, from the predation by a ciliate [11, 28]. In both examples, the mutants enjoy the advantage of being in the minority. This mechanism does not require the presence of a predator and can occur due to a large number of factors. For example, a mutant with chiral motility may spatially segregate from the rest of the population and thereby escape from an intense competition for resources [65].

Our main finding is that selection for chirality can also be mediated by the formation of non-trivial spatial patterns. Mismatch in the chiral bias makes cells move towards each other near in-flow boundaries and away from each other near out-flow boundaries. As a result, the colony edge becomes populated with bulges and dips, which grow over time and alter the relative abundance of the strains. One consequence of these dynamics is that it pays off to be different from the majority of the population: A mutant with the opposite handedness can invade when rare and stably coexist with the ancestor due to negative frequency-dependent selection. For strains with the same handedness, the more chiral strain typically wins the competition because it creates a one-sided bulge that overgrows the less chiral strain. Thus, we identified a distinct selection mechanism that can explain both the evolution toward stronger chirality and sudden reversal of handedness. The predicted effects of chirality are observable even in the presence of moderate growth rate differences and can be tested experimentally by comparing the competition between cells with different chirality to our predictions for colony shape and composition.

Selection for chirality could also come from the indirect benefits of the emergent spatial pattern. One possibility is that pointed bulges might facilitate the invasion of host tissue or other environments. The other possibility is that strain intermixing could promote social interactions that rely on cell contact or the exchange of diffusible metabolites. We found that intermixing between the strains with opposite handedness is stable only when their chiralities exceed a certain threshold. Below this threshold, genetic drift creates macroscopic sectors that grow over time and spatially segregate the strains. As a result of this process, social interactions are either suppressed or completely abolished [31–34].

All of our results can be explained by a simple effective theory that describes population dynamics in terms of colony shape and composition. This description is simpler and much more intuitive than the full two-dimensional growth encoded by reaction-diffusion equations or other mechanistic models. Therefore, our theory could provide a valuable framework to study competition and cooperation in compact aggregates such as microbial colonies and cancer tumors.

Shape undulations inevitably occur when aggregates contain strains that grow at different rates [35, 66]. So far, most theoretical studies have neglected this complexity and assumed that colonies have a flat front [31, 34, 41]. Front undulations, however, are known to profoundly change the nature of competition, for example, by allowing the regions with cooperating strains to overgrow the regions where cooperation has been lost [66, 67]. Our theory is an important first step towards understanding this interplay between evolution in compact aggregates and their shape.

The effective theory also provides an interesting extension of the KPZ equation to systems that break the mirror symmetry. Such symmetry breaking could occur in a variety of systems within the KPZ universality class, for example, during the simultaneous deposition of two homophilic molecules with opposite handedness.

In summary, we have identified a new mechanism of selection for chirality and developed a theory to explain it. Our findings describe the chirality of cells while most of the previous work focused on the emergence of homochirality in biological molecules [4–6, 19, 20]. Unlike the frozen homochirality of nucleotides and amino acids, the chirality of cells continues to evolve, often on the time scale of a few generations [11, 28–30]. Our work suggests that some changes in cellular chirality could be adaptive and, therefore, deserve further study.

## Methods

Lattice-based simulations were performed on a two-dimensional rectangular grid with periodic boundary conditions. The lattice spacings Δ*x* and Δ*y* were both set to 1. The length of each time step Δ*t* was set to 1 as well. Each time step, we first performed a deterministic update to account for growth and migration and then performed a stochastic update to account for demographic fluctuations and genetic drift. To speed up simulations, only a small rectangular region at the expanding edge was updated while the bulk remained frozen. This did not not affect the results since both migration and growth are zero in the colony bulk. The heterozygosity reported in Fig 8, was calculated within this rectangular region at each time step.

### Deterministic update

During the deterministic update, we computed an auxiliary quantity *ρ*^(*α*)^ equal to the expected value of *n*^(*α*)^ at the next time step:

$$\begin{array}{c} \rho^{\left(\alpha \right)}\left(t,x,y\right)=n^{\left(\alpha \right)}\left(t,x,y\right)+G^{\left(\alpha \right)}\left(t,x,y\right)\Delta t+M^{\left(\alpha \right)}\left(t,x,y\right)\Delta t. \end{array}$$ (6)

The growth term *G* describes the increase in the population density due to logistic growth:

$$\begin{array}{c} G^{\left(\alpha \right)}\left(t,x,y\right)=g^{\left(\alpha \right)}n^{\left(\alpha \right)}\left(t,x,y\right)(1-\frac{n(t,x,y)}{N}), \end{array}$$ (7)

where *n* = *n*^(1)^ + *n*^(2)^ is the total population size, and *N* is the carrying capacity. The growth rates *g*^(*α*)^ were typically the same for the two strains.

The migration term *M* describes the change in the population size due to migration:

$$\begin{array}{cc} M^{\left(\alpha \right)}\left(t,x,y\right)= & -m_{\left(x,y)\to (x+\Delta x,y\right)}^{\left(\alpha \right)}-m_{\left(x,y)\to (x,y+\Delta y\right)}^{\left(\alpha \right)}-m_{\left(x,y)\to (x-\Delta x,y\right)}^{\left(\alpha \right)}-m_{\left(x,y)\to (x,y-\Delta y\right)}^{\left(\alpha \right)}\\ & +m_{\left(x+\Delta x,y)\to (x,y\right)}^{\left(\alpha \right)}+m_{\left(x,y+\Delta y)\to (x,y\right)}^{\left(\alpha \right)}+m_{\left(x-\Delta x,y)\to (x,y\right)}^{\left(\alpha \right)}+m_{\left(x,y-\Delta y)\to (x,y\right)}^{\left(\alpha \right)}, \end{array}$$ (8)

where $m_{\left(x_{1},y_{1})\to (x_{2},y_{2}\right)}^{\left(\alpha \right)}\Delta t$ is the expected number of migrants of strain *α* from the site at (*x*_1_, *y*_1_) into the site at (*x*_2_, *y*_2_). The first four terms in the equation describe migration out of the lattice site (*x*, *y*) and the last four terms describe the migration into the lattice site (*x*, *y*). Note that the number of cells leaving a particular site into the direction of its nearest neighbor is equal to the number of cells arriving into that neighboring site, i.e. migration conserves the number of cells.

The migration fluxes $m_{\left(x_{1},y_{1})\to (x_{2},y_{2}\right)}^{\left(\alpha \right)}$ were nonzero only between the four nearest neighbors and were defined as follows

$$\begin{array}{cc} & m_{\left(x,y)\to (x+\Delta x,y\right)}^{\left(\alpha \right)}=n^{\left(\alpha \right)}\left(t,x,y\right)(1-\frac{n(t,x+\Delta x,y)}{N})\times \\ & (m_{0}^{\left(\alpha \right)}+m_{s}^{\left(\alpha \right)}\frac{n(t,x,y)}{N}+m_{d}^{\left(\alpha \right)}\frac{n(t,x+\Delta x,y)}{N}+m_{l}^{\left(\alpha \right)}\frac{n(t,x,y+\Delta y)}{N}+m_{b}^{\left(\alpha \right)}\frac{n(t,x-\Delta x,y)}{N}+m_{r}^{\left(\alpha \right)}\frac{n(t,x,y-\Delta y)}{N}),\\ & m_{\left(x,y)\to (x,y+\Delta y\right)}^{\left(\alpha \right)}=n^{\left(\alpha \right)}\left(t,x,y\right)(1-\frac{n(t,x,y+\Delta y)}{N})\times \\ & (m_{0}^{\left(\alpha \right)}+m_{s}^{\left(\alpha \right)}\frac{n(t,x,y)}{N}+m_{d}^{\left(\alpha \right)}\frac{n(t,x,y+\Delta y)}{N}+m_{l}^{\left(\alpha \right)}\frac{n(t,x-\Delta x,y)}{N}+m_{b}^{\left(\alpha \right)}\frac{n(t,x,y-\Delta y)}{N}+m_{r}^{\left(\alpha \right)}\frac{n(t,x+\Delta x,y)}{N}),\\ & m_{\left(x,y)\to (x-\Delta x,y\right)}^{\left(\alpha \right)}=n^{\left(\alpha \right)}\left(t,x,y\right)(1-\frac{n(t,x-\Delta x,y)}{N})\times \\ & (m_{0}^{\left(\alpha \right)}+m_{s}^{\left(\alpha \right)}\frac{n(t,x,y)}{N}+m_{d}^{\left(\alpha \right)}\frac{n(t,x-\Delta x,y)}{N}+m_{l}^{\left(\alpha \right)}\frac{n(t,x,y-\Delta y)}{N}+m_{b}^{\left(\alpha \right)}\frac{n(t,x+\Delta x,y)}{N}+m_{r}^{\left(\alpha \right)}\frac{n(t,x,y+\Delta y)}{N}),\\ & m_{\left(x,y)\to (x,y-\Delta y\right)}^{\left(\alpha \right)}=n^{\left(\alpha \right)}\left(t,x,y\right)(1-\frac{n(t,x,y-\Delta y)}{N})\times \\ & (m_{0}^{\left(\alpha \right)}+m_{s}^{\left(\alpha \right)}\frac{n(t,x,y)}{N}+m_{d}^{\left(\alpha \right)}\frac{n(t,x,y-\Delta y)}{N}+m_{l}^{\left(\alpha \right)}\frac{n(t,x+\Delta x,y)}{N}+m_{b}^{\left(\alpha \right)}\frac{n(t,x,y+\Delta y)}{N}+m_{r}^{\left(\alpha \right)}\frac{n(t,x-\Delta x,y)}{N}), \end{array}$$ (9)

where the factors of *n*^(*α*)^ ensure that the number of migrants is proportional to the local abundance of the strain, and the factors of $1-\frac{n}{N}$ ensure that migration cannot occur into occupied lattice sites. As a result of these choices, the spatial distribution of the strains remains “frozen” behind the growing front just as in microbial colonies, where the growth in the bulk of the colony is suppressed. The last factor in each of the equations describes the dependence of the migration rates on the local population population density and its spatial gradients; this can be seen by expanding population densities into Taylor series.

**Fig 9:**
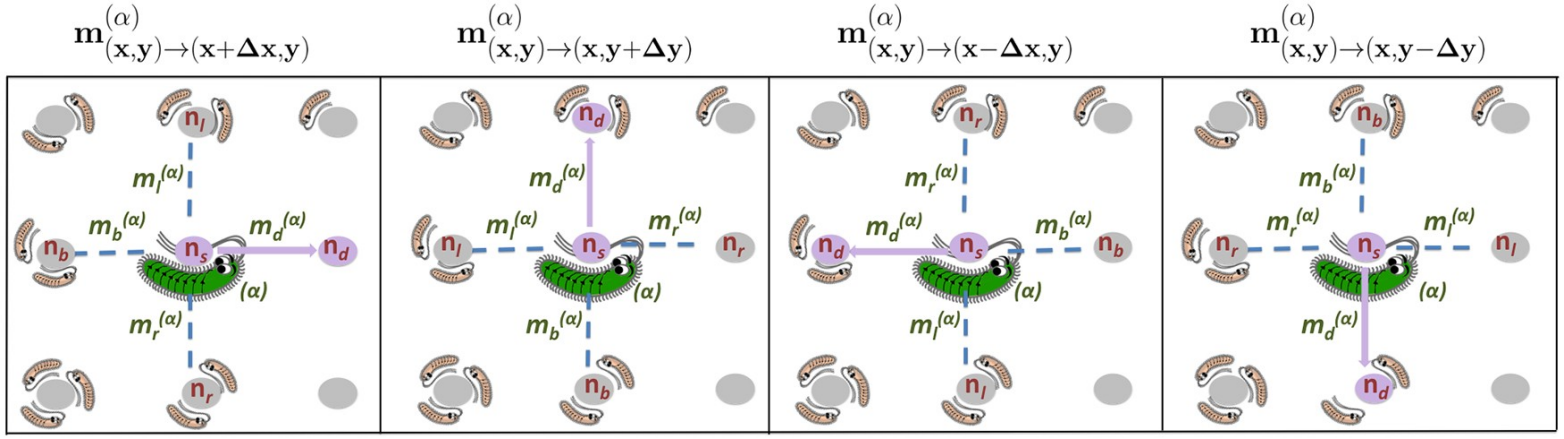
Isotropic, but chiral migration in a lattice-based model. The panels illustrate the computation of migration fluxes $m_{\left(x_{1},y_{1})\to (x_{2},y_{2}\right)}^{\left(\alpha \right)}$ given by Eq (9) for different orientations of the migration direction relative to the lattice. Note that the choice of the migration coefficients is always made relative to the direction of migration and not relative to the coordinate system.

Note that our definitions preserve the equivalence of all four lattice direction because the migration coefficients are chosen according to the position of the lattice sites relative to the direction of the migration rather than relative to a particular lattice direction; see Fig 9. To emphasize this fact, we use the index labels that refer to source site, destination site, left site, back site, and right site—all specified with respect to the migration direction. For simplicity, we limited the dependence on *n* to the lowest order of the Taylor expansion that is sufficient to produce chiral growth.

The relationship between the model parameters and the coefficients in the continuum description is provided below:

$$\begin{array}{cc} & D^{\left(\alpha \right)}\left(n\right)=[m_{0}^{\left(\alpha \right)}+\frac{n}{N}(m_{s}^{\left(\alpha \right)}+m_{d}^{\left(\alpha \right)}+m_{l}^{\left(\alpha \right)}+m_{b}^{\left(\alpha \right)}+m_{r}^{\left(\alpha \right)})](1-\frac{n}{N})\frac{\Delta x^{2}}{\Delta t}\\ & S^{\left(\alpha \right)}\left(n\right)=[2(m_{b}^{\left(\alpha \right)}-m_{d}^{\left(\alpha \right)})(1-\frac{n}{N})+(m_{0}^{\left(\alpha \right)}+m_{s}^{\left(\alpha \right)}+m_{d}^{\left(\alpha \right)}+m_{l}^{\left(\alpha \right)}+m_{b}^{\left(\alpha \right)}+m_{r}^{\left(\alpha \right)})]\frac{\Delta x^{2}}{\Delta t}\\ & A^{\left(\alpha \right)}\left(n\right)=2(m_{l}^{\left(\alpha \right)}-m_{r}^{\left(\alpha \right)})(1-\frac{n}{N})\frac{\Delta x^{2}}{\Delta t} \end{array}$$ (10)

From Eq (10), it is clear that *A*^(*α*)^ depends on $m_{l}^{\left(\alpha \right)}-m_{r}^{\left(\alpha \right)}$ while *D*^(*α*)^ depends on $m_{l}^{\left(\alpha \right)}+m_{r}^{\left(\alpha \right)}$. Thus, one can vary the chirality of a strain without affecting its motility. We used this freedom to isolate the effects of chirality from other components of strain fitness in most of our simulations by keeping $m_{l}^{\left(\alpha \right)}+m_{r}^{\left(\alpha \right)}$ fixed.

### Stochastic update

The stochastic update consisted of two rounds of binomial sampling.

The first round accounted for the demographic fluctuations in the total population size. We drew *n*(*t* + Δ*t*, *x*, *y*) from a binomial distribution with *N* trials and (*ρ*^(1)^(*t*, *x*, *y*) + *ρ*^(2)^(*t*, *x*, *y*))/*N* probability of success. This procedure ensures that (i) the expectation value of *n* is consistent with the deterministic dynamics, (ii) the size of a typical fluctuation scales as $\sqrt{n}$ for *n* ≪ *N*, and (iii) the population size never exceeds the carrying capacity *N*.

The second round accounted for genetic drift. We drew *n*^(1)^(*t* + Δ*t*, *x*, *y*) from a binomial distribution with *n*(*t* + Δ*t*, *x*, *y*) trials and *ρ*^(1)^(*t*, *x*, *y*)/(*ρ*^(1)^(*t*, *x*, *y*) + *ρ*^(2)^(*t*, *x*, *y*)) probability of success. The abundance of the other strain was set to *n*^(2)^(*t* + Δ*t*, *x*, *y*) = *n*(*t* + Δ*t*, *x*, *y*) − *n*^(1)^(*t* + Δ*t*, *x*, *y*). This stochastic update does not change the relative fractions of the two strains on average, and the typical fluctuation in the relative abundance of the strains scales as $\sqrt{n}$.

### Off-lattice simulations

To ensure that our results do not arise because of the lattice effects, we developed off-lattice simulations of our reaction-diffusion model. In these simulations, cells reproduced stochastically depending on the local population density and performed short-range jumps. The magnitude of the jump was controlled by the population density and the direction of the jump depended on the local density gradient and the chirality of the cell. The functional forms of the growth rates and the jump kernels are provided in the SI.

Off-lattice simulations confirmed the predictions of our theory and lattice-based simulations. Specifically, we observed stabilizing selection and the formation of bulges between strains with opposite handedness. These results are shown in the SI. Because of computational efficiency, most of the analysis was carried out using lattice-based simulations.

## Supporting information

S1 Text Derivation of the effective theory and analytical predictions. The text formulates the reaction-diffusion model, and derives the effective theory in two ways: from the reaction-diffusion model and from phenomenological considerations. It provides analytical solutions for the behavior of in-flow and out-flow boundaries, the shapes of bulges and dips, and natural selection from the coupling of front shape to strain dynamics. The text also describes the procedure for off-lattice simulations.
(PDF)

S1 FigQuantitative test of Eq (5), the relationship between ${\bar{f}}_{eq}$ and f*.**(A)** shows the shape (shape preserving spline) of an asymmetric bulge from a simulation with f* = 0.75. The red lines are the best fit of the two slopes of the bulge. From these slopes, we obtained $\frac{\alpha }{D_{h}}=0.080$ and $\frac{\beta }{v_{0}}=0.068$ using Eq. (S55); the values of these parameters are averages over all runs with f* ≠ 0.5. **(B)** shows ${\bar{f}}_{eq}$ from simulations (dots) and the theoretical prediction (line) from Eq (5) in the main text and the estimated values of the parameters. The predicted slope equals 0.856 and is quite close to 0.82 ± 0.02, which is the slope obtained by ordinary least squares regression (not shown). The root mean square deviation between the theory and the simulations is 0.01. Here, *m*_0_ = *m*_*s*_ = *m*_*b*_ = *m*_*d*_ = 0, *g* = 0.1, *m*_*l*_ + *m*_*r*_ = 0.01 for both strains. Simulations started from two separate domains on a lattice of 1200x14000 sites with *N* = 800. We ensured that the simulations reached steady state by starting runs from an initial fractions of 0.25, 0.5 and 0.75. Error bars (s.e.m) were estimated from a set of 18 runs with 6 starting from each initial fraction.(TIF)

S2 FigThe size of the coexistence region depends on model parameters.This figure is the same as Fig 7B, but for different model parameters. In comparison with the figure in the main text, the coexistence region is smaller, and the slope of ${\bar{f}}_{eq}$ is steeper. The green line is the least squares fit to the simulation data (dots); the slope is 1.45, and *R*^2^ = 0.997. The solid part of the line spans the data points with ${\bar{f}}_{eq}\in (0,1)$ that were used in the fit. The dashed part extends this dependence to the entire region of possible ${\bar{f}}_{eq}$. The black dashed line marks the unit slope. Here, *m*_0_ = 0.01, *m*_*s*_ = *m*_*b*_ = *m*_*d*_ = 0, *g* = 0.1, *m*_*l*_ + *m*_*r*_ = 0.1 for both strains. Simulations started from well mixed conditions on a lattice of width 300 sites with *N* = 200. The simulation time was chosen to ensure that the same steady state was approached starting from initial conditions that are both above and below ${\bar{f}}_{eq}$.(TIF)

S3 Fig Genetic drift, but not chirality control the boundary width between identical strains. **(A)** shows that domain boundaries between strains with equal chirality become wider for larger *N* (weaker genetic drift); the dependence is approximately linear in agreement with Ref. [63]. **(B)** In contrast, chirality has no detectable effect on the boundary width. The rest of the panels show the spatial patterns used to reach these conclusions. The boundary width was computed as the local heterozygosity, 2〈*f*(1 − *f*)〉, summed over the entire width of the simulation and averaged over *y* ∈ (2000, 4000). Simulations were performed on a lattice of 1000x4000 sites with *m*_0_ = *m*_*s*_ = *m*_*b*_ = *m*_*d*_ = 0, *g* = 0.1.
(TIF)

S4 Fig Mixing transitions at different values of relative chirality. **(A), (B), (C)** show demixed phases for strong genetic drift and varying values of f*. **(D), (E)** shows dissolution of a boundary and the establishment of the intermixed phase for weak genetic drift and two values of f*. **(F)** shows the competition between a chiral and a non-chiral strain for weak genetic drift. The boundary is much wider than in (C), but no intermixed phase is established because the non-chiral strain is outcompeted. Here, *m*_0_ = *m*_*s*_ = *m*_*b*_ = *m*_*d*_ = 0, *g* = 0.1, *m*_*l*_ + *m*_*r*_ = 0.1 for both strains. Simulations were carried out on a lattice of 600x3000 sites.
(TIF)

S5 FigChirality differences, but not genetic drift control the time scale of selection.**(A)** shows that the fixation time of the chiral strain decreases with the magnitude of its chirality. Similarly, **(B)** demonstrates that stronger chirality results in shorter equilibration times for two strains with opposite chirality. **(C)**, and **(D)** show that genetic drift does not affect the time scale of selection. Here, *m*_0_ = *m*_*s*_ = *m*_*b*_ = *m*_*d*_ = 0, *g* = 0.1, *m*_*l*_ + *m*_*r*_ = 0.1 for both strains. In (A) and (B), simulations were started from well-mixed initial conditions with *N* = 400 on a lattice of 500x6000 and 1000x1200 sites respectively. Simulations started from two demixed domains with $m_{l}^{\left(1\right)}=0.09$, $m_{r}^{\left(1\right)}=0.01$, $m_{l}^{\left(2\right)}=0.05$, $m_{r}^{\left(2\right)}=0.05$, on a lattice of 500x7500 sites in (C), and $m_{l}^{\left(1\right)}=0.09$, $m_{r}^{\left(1\right)}=0.01$, $m_{l}^{\left(2\right)}=0.01$, $m_{r}^{\left(2\right)}=0.09$ on a lattice of 500x3000 sites in (D).(TIF)

S6 Fig Effects of chirality on the expansion velocity. In panel **(A)**, we demonstrate that a change in chirality does not produce a change in the expansion velocity of a strain grown in isolation when *m*_*l*_ + *m*_*r*_ is kept fixed. **(B)** shows that the expansion velocity increases, but only slightly, when two strains with opposite handedness expand together. Here, *m*_0_ = *m*_*s*_ = *m*_*b*_ = *m*_*d*_ = 0, *g* = 0.1, *m*_*l*_ + *m*_*r*_ = 0.1 for both strains. Simulations started from well-mixed conditions on a lattice of 200x1000 sites with *N* = 200. Error bars (s.e.m) were estimated from 4 identical runs and velocity is measured in units of lattice spacing.
(TIF)

S7 Fig Chirality affects competition in off-lattice simulations. **(A)** shows the emergence of a bulge between two oppositely chiral strains. **(B)** shows the stabilizing selection between two oppositely chiral strains similar to Fig 3C in the main text. The species fractions were averaged over 6 runs. Parameters were *N* = 6, *A*^(1)^ = 10, *A*^(2)^ = −10, *g*^(1)^ = *g*^(2)^ = 0.2 for both figures, and *W* = 200, *μ* = 1.0 in (A) and *W* = 500, *μ* = 0.5 in (B). Distances were rescaled by a factor of 100, similar to simulations on the lattice in the text.
(TIF)
