# Supplementary material for: Chirality provides a direct fitness advantage and facilitates intermixing in cellular aggregates
Source: PLoS Comput Biol. 2018 Dec 27;14(12):e1006645. doi: 10.1371/journal.pcbi.1006645 (PMC6307711; doi:10.1371/journal.pcbi.1006645)
Supplement: S1 Text — The text formulates the reaction-diffusion model, and derives the effective theory in two ways: from the reaction-diffusion model and from phenomenological considerations. It provides analytical solutions for the behavior of in-flow and out-flow boundaries, the shapes of bulges and dips, and natural selection from the coupling of front shape to strain dynamics. The text also describes the procedure for off-lattice simulations. (PDF) [file pcbi.1006645.s001.pdf]

Supporting Information: Chirality provides a direct fitness advantage and facilitates intermixing in cellular aggregates

Ashish B. George[1], Kirill S. Korolev[2†]

1 Department of Physics, Boston University, Boston, MA 02215, USA
2 Department of Physics and Graduate Program in Bioinformatics,Boston University, Boston, MA 02215, USA

* ashish.b.george@gmail.com †korolev@bu.edu

Contents

S1 Formulation of the reaction-diffusion model **2**

S2 Derivation of the effective theory of front shape and composition **3**

S3 Derivation of the effective theory from phenomenological considerations **8**

S4 Deterministic behavior of in-flow and out-flow boundaries in flat fronts **12**

S5 Deterministic solution for bulge and dip shape near a sharp boundary **14**

S6 Natural selection due to the influence of front shape on the dynamics of $f(t,x)$ **20**

S7 Off-lattice simulations **22**

S1 Formulation of the reaction-diffusion model

The goal of this section is to formulate a reaction-diffusion model that describes competition between two chiral strains in growing colonies. Quite generally, a reaction-diffusion model can be stated as follows

$$\frac{\partial n^{(\alpha)}}{\partial t} = g^{(\alpha)} n^{(\alpha)} - \boldsymbol{\nabla} \cdot \boldsymbol{J}^{(\alpha)}, \tag{S1}$$

where $n^{(\alpha)}(t, x, y)$ is the population density of the strain of type α at time t and position (x, y); $g^{(\alpha)}$ is the growth rate; and $J^{(\alpha)}$ is the population flux due to movement. Both $g^{(\alpha)}$ and $J^{(\alpha)}$ could depend on the population densities of all strains, but not on t, x, or y because we assume spatial and temporal homogeneity. Here and below, bold symbols denote two dimensional vectors and $\boldsymbol{\nabla}$ denotes the gradient operator.

The chirality of the strains manifests through their active or passive movement and is encoded in $\boldsymbol{J}^{(\alpha)}$. Assuming that $n^{(\alpha)}$ are the only relevant dynamical variables, one has only two vectors that can determine the orientation of $\boldsymbol{J}^{(\alpha)}$. These are $\boldsymbol{\nabla}_i n^{(1)}$ and $\boldsymbol{\nabla}_i n^{(2)}$ or equivalently the gradient of the total population density $n = n^{(1)} + n^{(2)}$ and the gradient of the population density of a specific strain. Thus, to the first order in the gradient expansion one can write the population fluxes as

$$\boldsymbol{J}_i^{(\alpha)} = -D^{(\alpha)} \boldsymbol{\nabla}_i n^{(\alpha)} - n^{(\alpha)} \sum_j \left(S^{(\alpha)} \delta_{ij} - A^{(\alpha)} \epsilon_{ij} \right) \boldsymbol{\nabla}_j n, \tag{S2}$$

where the indexes denote the Cartesian components of vectors; δ_{ij} is the unit tensor; and ϵ_{ij} is the totally antisymmetric tensor, also known as Levi-Civita symbol. The first term in the equation accounts for diffusion-like movement. The second term describes the advection of the strain density by local motion within the aggregate. This motion arises due to gradients of mechanical pressure, chemotaxis, and other factors. The direction of the advection velocity is determined by the gradient of the total population density. The term proportional to $S^{(\alpha)}$ describes the flux along the gradient, which is the only possibility for non-chiral organisms. The term proportional to $A^{(\alpha)}$ describes the motion perpendicular to $\boldsymbol{\nabla} n$ due to chirality. The above decomposition of the drift term into symmetric and antisymmetric parts is always possible because δ_{ij} and ϵ_{ij} are the only rotationally invariant tensors in two dimensions. Note that we do not include a separate term proportional to $\epsilon_{ij} \boldsymbol{\nabla}_j n^{(\alpha)}$ because its contribution to Eq. (S1) is indistinguishable from the contribution of the term proportional to $A^{(\alpha)}$. In the following, we refer to $A^{(\alpha)}$ as the microscopic "chiralities" of the strains. The sign of these chiralities describes the overall chirality of the strain: positive for strains that tend to move to the left relative to the expansion direction and negative for strains that tend to move to the right relative to the expansion direction. The magnitudes of the chiralities reflect the strength of the movement biases.

Since our primary goal is to understand the effects chirality, we exclude from consideration all differences between the strains that are possible even when the strains have the same chirality. In particular, we assume that $g^{(\alpha)}$, $D^{(\alpha)}$, $S^{(\alpha)}$ are equal for the two strains and depend only on the total population density. The coefficients describing chirality could be different between the two strains, but we still assume that $A^{(\alpha)}$ depend only on n. Note that local interaction between the chiral strains, e.g. via alignment, could results in the dependence of $D^{(\alpha)}$, $S^{(\alpha)}$, and $A^{(\alpha)}$ on the relative fractions of the species. It is easy to check, however, that this dependence generates terms in Eq. (S1) that are invariant under mirror symmetry. When such terms arise due to chirality, they must scale at least quadratically in the difference between the chiralities of the two strains because this difference changes sign under the application of the mirror-symmetry transformation.[1] Further, the effects of mirror-symmetric terms are less interesting because they could arise even in the absence of chirality.

[1] We discuss this point further in the section devoted to the phenomenological derivation of the effective equations for the shape and composition of the edge of a growing colony.

After the simplifications just described, the equation governing population dynamics reads

$$\frac{\partial n^{(\alpha)}}{\partial t} = g(n)n^{(\alpha)} + \boldsymbol{\nabla}\left(D(n)\boldsymbol{\nabla} n^{(\alpha)}\right) + \boldsymbol{\nabla}\left(n^{(\alpha)}S(n)\boldsymbol{\nabla} n\right) - A^{(\alpha)}(n)\sum_{ij}\epsilon_{ij}(\boldsymbol{\nabla}_i n^{(\alpha)})(\boldsymbol{\nabla}_j n). \tag{S3}$$

Note that $A^{(\alpha)}(n)$ can be taken outside of the derivatives because $\sum_{ij}\epsilon_{ij}\boldsymbol{\nabla}_i n\boldsymbol{\nabla}_j n = 0$.

Althoug the analysis can be carried out for arbitrary $g(n)$, $D(n)$, $S(n)$, and $A^{(\alpha)}(n)$. We will make a few assumptions that significantly simplify the discussion and make it easier to connect the theory to experimental results shown in Fig. 1 in the main text. First, we assume that as the population grows it reaches a stable state at $n = N$, which correspond to the carrying capacity of the population. Mathematically, this assumption can be stated as $g(N) = 0$ and $\left.\frac{dg}{dn}\right|_{n=N} < 0$. Second, we assume that all migration ceases once the population reaches the carrying capacity. That is $D(N)$, $S(N)$, and $A^{(\alpha)}(N)$ are zero. This assumption reflects the fact that no dynamics occur behind the expansion front in the experiments shown in Fig.1 in the main text. When this assumption does not hold, the results derived below apply just at the front of the population and do not describe the dynamics in the bulk of the colony. The third assumption is that the population expands as a "pushed" reaction-diffusion wave *sensu* Ref. [1]. This means that the expansion velocity is greater than the expansion velocity obtained by linearizing Eq. (S4) for small n, which is known as the Fisher or linear spreading velocity [1]. Pushed waves occurs when at least one function out of $D(n)$, $S(n)$, and $g(n)$ exhibits a substantial increase with n relative to its value at $n = 0$. Because these functions vanish at $n = N$, at least one of the functions must be non-monotonic in n.

The main reason why we assume that the expansions are pushed is that Eq. (S3) predicts no chiral phenomena for pulled waves. Indeed, the dynamics of pulled waves are described by the equation linearized around $n = 0$, in which the chiral term vanishes because it is quadratic in $\boldsymbol{\nabla} n$.

To understand the competition between the strains, it is convenient to recast Eq. (S3) in terms of the total population density and the relative fraction of the first strain $f = n^{(1)}/n$. This change of variables results in the following equations

$$\frac{\partial n}{\partial t} = g(n)n + \boldsymbol{\nabla}\left[(D(n) + nS(n))\boldsymbol{\nabla} n\right] - \left[A^{(1)}(n) - A^{(2)}(n)\right] n\sum_{ij}\epsilon_{ij}(\boldsymbol{\nabla}_i f)(\boldsymbol{\nabla}_j n), \tag{S4}$$

and

$$\begin{aligned}\frac{\partial f}{\partial t} =& \boldsymbol{\nabla}\left[D(n)\boldsymbol{\nabla} f\right] + \left[\frac{2D(n)}{n} + S(n)\right](\boldsymbol{\nabla} n)(\boldsymbol{\nabla} f) \\ &- \left[A^{(1)}(n) - A^{(2)}(n)\right]\left[\frac{A^{(1)}(n)}{A^{(1)}(n) - A^{(2)}(n)} - f\right]\sum_{ij}\epsilon_{ij}(\boldsymbol{\nabla}_i f)(\boldsymbol{\nabla}_j n).\end{aligned} \tag{S5}$$

S2 Derivation of the effective theory of front shape and composition

In this section, we replace the detailed model of growth in two spatial dimensions by an approximate model in terms of the shape and composition of the population front. The motivation for this approximation is

clear: Away from the edge, the population densities are either zero or stationary because n is at the carrying capacity. The approach presented below is a type of dimensional reduction that eliminates one of the spatial variables and is reminiscent of the moving-boundary approximation developed in other contexts [1]. The final result is an effective description in terms of the position of the population front and the composition of the population at the front.

For simplicity, we consider the colony front that is mostly parallel to the x-axis so that the shape of the front can be described by its y coordinate as a function of x. We denote this dependence as $h(t, x)$ and refer to front position as height in analogy with surface growth phenomena. The composition of the population at the growing edge is described by the fraction of the first strain $f(t, x)$. Below, we derive the equations governing the dynamics of $h(t, x)$ and $f(t, x)$ starting from Eqs. (S4) and (S5). The main two assumptions underlying this derivation is that the front undulations are small and that there is little variation in f across the thickness of the colony edge. These assumptions require that the expansion is pushed and that the difference in the chiralities of the two strains is small.

Equation for front shape, $h(t, x)$

The equation for $h(t, x)$ follows from Eq. (S4), which we re-write as

$$\frac{\partial n}{\partial t} = g(n)n + \boldsymbol{\nabla}\left(D_e \boldsymbol{\nabla} n\right) - \mathcal{A}\sum_{ij} \epsilon_{ij}(\boldsymbol{\nabla}_i f)(\boldsymbol{\nabla}_j n) \tag{S6}$$

by introducing a more compact notation: $D_e = D + nS$ and $\mathcal{A} = (A^{(1)} - A^{(2)})n$.

To carry out the dimensional reduction described above, we assume that $n(t, x, y) = n(\zeta)$, where ζ is the distance between (x, y) and the line defined by the new dynamical variable $h(t, x)$. The new variable $h(t, x)$ represents the y coordinate of the population front for a given x and could, for example, be defined such that $n(t, x, h(t, x)) = 1/2 \max_{(x,y)}\{n(t, x, y)\}$. For a flat front parallel to the x-axis, $\zeta = y - h(t, x)$, but for a tilted front ζ is smaller and is given by the projection of $y - h$ on the direction perpendicular to the front. In the following, we assume that $\frac{\partial h}{\partial x} \ll 1$ and keep only the terms up to the second order in $\frac{\partial}{\partial x}$. Under these assumptions, the tilt of the front is given by $\frac{\partial h}{\partial x}$ and the expression for ζ reads

$$\zeta = [y - h(t, x)]\left[1 - \frac{1}{2}\left(\frac{\partial h}{\partial x}\right)^2\right]. \tag{S7}$$

We now evaluate all the terms that enter Eq. (S6) up to the second order in $\frac{\partial}{\partial x}$:

$$
\begin{aligned}
\frac{\partial n}{\partial t} &= -\frac{dn}{d\zeta}\frac{\partial h}{\partial t}\left[1-\frac{1}{2}\left(\frac{\partial h}{\partial x}\right)^2\right],\\
\boldsymbol{\nabla} n &= \frac{dn}{d\zeta}\left[1-\frac{1}{2}\left(\frac{\partial h}{\partial x}\right)^2\right]\left(-\frac{\partial h}{\partial x},1\right),\\
(\boldsymbol{\nabla} n)^2 &= \left(\frac{dn}{d\zeta}\right)^2,\\
\boldsymbol{\nabla}^2 n &= \frac{d^2 n}{d\zeta^2}-\frac{dn}{d\zeta}\frac{\partial^2 h}{\partial x^2},\\
\sum_{ij}\epsilon_{ij}(\boldsymbol{\nabla}_i f)(\boldsymbol{\nabla}_j n) &= \frac{\partial f}{\partial x}\frac{dn}{d\zeta},
\end{aligned}
\tag{S8}
$$

where, in the last expression, we assumed that f remains constant in the direction perpendicular to the front. Upon substituting Eq. (S8) into Eq. (S6), we obtain

$$
\frac{d}{d\zeta}\left(D_e\frac{dn}{d\zeta}\right)+\frac{dn}{d\zeta}\frac{\partial h}{\partial t}\left[1-\frac{1}{2}\left(\frac{\partial h}{\partial x}\right)^2\right]+gn-\frac{dn}{d\zeta}\left(D_e\frac{\partial^2 h}{\partial x^2}+\mathcal{A}\frac{\partial f}{\partial x}\right)=0. \tag{S9}
$$

To understand this equation, let us first consider a flat front parallel to the x-axis ($\frac{\partial h}{\partial x}=0$) with no spatial variation in f. Such a front moves along the y-axis with a constant velocity v_0, i.e. $\frac{\partial h}{\partial t}=v_0$. Hence, Eq. (S9) reduces to

$$
\frac{d}{d\zeta}\left(D_e\frac{dn}{d\zeta}\right)+v_0\frac{dn}{d\zeta}+gn=0, \tag{S10}
$$

which is the standard equation for the population density profile of a reaction-diffusion wave. The solution of this equation has to satisfy three constraints: $\lim_{\zeta\to-\infty} n=0$, $\lim_{\zeta\to+\infty} n=N$, and $n(0)=N/2$, which eliminates the translational degree of freedom by specifying the position of a particular population density. Since Eq. (S10) is second order, the three constraints cannot be satisfied simultaneously for an arbitrary v_0. Thus, Eq. (S10) determines both the profile shape $n(\zeta)$ and the expansion velocity v_0. We note that this simple argument applies only to pushed waves, and additional analysis is needed to determine the expansion velocity of pulled waves [1–3].

We now return to the full Eq. (S9) and analyze it in the limit of $\frac{\partial h}{\partial t}$, $\frac{\partial h}{\partial x}$, $\frac{\partial^2 h}{\partial x^2}$, and $\frac{\partial f}{\partial x}$ being approximately constant. The first three terms in Eq. (S9) are then equivalent to Eq. (S10) while the remaining terms constitute a small perturbation that depends on n. This perturbation leads to a correction to the expansion velocity, which can be evaluated using the results from Refs. [3–6]. The expression for the expansion velocity can then be equated to the coefficient in front of $\frac{dn}{d\zeta}$ in the third term of Eq. (S9), which leads to

$$
\frac{\partial h}{\partial t}\left(1-\frac{1}{2}\left(\frac{\partial h}{\partial x}\right)^2\right)=v_0+\frac{\int_{-\infty}^{+\infty}\left[D_e(n_0(\zeta))\frac{\partial^2 h}{\partial x^2}+\mathcal{A}(n_0(\zeta))\frac{\partial f}{\partial x}\right]D_e(n_0(\zeta))\left(\frac{dn_0(\zeta)}{d\zeta}\right)^2 e^{v_0\int_0^\zeta\frac{d\tilde{\zeta}}{D_e(n_0(\tilde{\zeta}))}}d\zeta}{\int_{-\infty}^{+\infty}D_e(n_0(\zeta))\left(\frac{dn_0(\zeta)}{d\zeta}\right)^2 e^{v_0\int_0^\zeta\frac{d\tilde{\zeta}}{D_e(n_0(\tilde{\zeta}))}}d\zeta}, \tag{S11}
$$

where $n_0(\zeta)$ is the density profile satisfying Eq. (S10). Since $\frac{\partial^2 h}{\partial x^2}$ and $\frac{\partial f}{\partial x}$ do not depend on ζ, they can be taken outside of the integrals and we obtain the following equation for $h(t,x)$:

$$\frac{\partial h}{\partial t} = v_0 + \frac{v_0}{2}\left(\frac{\partial h}{\partial x}\right)^2 + D_h \frac{\partial^2 h}{\partial x^2} + \alpha \frac{\partial f}{\partial x}, \tag{S12}$$

where

$$D_h = \frac{\int_{-\infty}^{+\infty} D_e^2(n_0(\zeta)) \left(\frac{dn_0(\zeta)}{d\zeta}\right)^2 e^{v_0 \int_0^{\zeta} \frac{d\tilde{\zeta}}{D_e(n_0(\tilde{\zeta}))}} d\zeta}{\int_{-\infty}^{+\infty} D_e(n_0(\zeta)) \left(\frac{dn_0(\zeta)}{d\zeta}\right)^2 e^{v_0 \int_0^{\zeta} \frac{d\tilde{\zeta}}{D_e(n_0(\tilde{\zeta}))}} d\zeta}, \tag{S13}$$

and

$$\alpha = \frac{\int_{-\infty}^{+\infty} \left\{[A^{(1)}(n_0(\zeta)) - A^{(2)}(n_0(\zeta))]n_0(\zeta)\right\} D_e(n_0(\zeta)) \left(\frac{dn_0(\zeta)}{d\zeta}\right)^2 e^{v_0 \int_0^{\zeta} \frac{d\tilde{\zeta}}{D_e(n_0(\tilde{\zeta}))}} d\zeta}{\int_{-\infty}^{+\infty} D_e(n_0(\zeta)) \left(\frac{dn_0(\zeta)}{d\zeta}\right)^2 e^{v_0 \int_0^{\zeta} \frac{d\tilde{\zeta}}{D_e(n_0(\tilde{\zeta}))}} d\zeta}. \tag{S14}$$

Equation (S12) is the main result of this subsection.

The first three terms on the right hand side of Eq. (S12) represent the standard KPZ equation of surface growth [7–9]. The first term accounts for the growth of a flat surface. The second term accounts for the effects of front tilt: Displacement of a tilted interface along its normal by dl increases the height by $dl\left(1 + \frac{1}{2}\left(\frac{\partial h}{\partial x}\right)^2\right)$. The third term accounts for the lateral diffusion in the context of surface-deposition models or for the effect of front curvature on the expansion velocity in the context of reaction-diffusion waves. A convex front expands more slowly compared to a flat front because it needs to cover a large area in order to advance by the same distance.

The remaining term on the right hand side of Eq. (S12) reflects the differences in the chiralities of the strains. It explicitly breaks the reflection symmetry $x \to -x$ and, therefore, must be absent in a model of competition between non-chiral strains. The main effect of this new term is to promote growth at the boundaries where the strains move towards each other and suppress growth at the boundaries where the strains move away from each other. To see this, let f denote the fraction of the strain that has a stronger left-moving bias. With this choice, $A^{(1)} > A^{(2)}$ (see Eq. (S2)), and, therefore, $\alpha > 0$. The extra growth due to $\alpha\frac{\partial f}{\partial x} > 0$ then occurs only when the fraction of the left-moving strain increases with x, i.e. when the left-moving strain is to the right of the right-moving strain. For this arrangement, the bias of the strains brings them towards each other, which is precisely the condition for extra growth stated above. We examine the effects of this extra growth quantitatively in a separate section below.

Equation for front composition, $f(t, x)$

The equation for $f(t, x)$ follows from Eq. (S5) for $f(t, x, y)$. Note that we denote these two quantities by the same symbol and distinguish them by specifying their arguments explicitly. In the following, we use exclusively $f(t, x, y)$ until we derive the effective equation for the dynamics of the species fractions at the front.

We begin by changing variables from t, x, and y to τ, η, and ζ. The variable ζ denotes the distance between (x, y) and $h(t, x)$ as before. The variable η specifies the position along the front and is defined as a curvilinear coordinate orthogonal to ζ. The variable τ equals to t. We introduce this new time variable explicitly to emphasize the fact that $\frac{\partial}{\partial t} \neq \frac{\partial}{\partial \tau}$ because the change of variables depends on time through $h(t, x)$.

In the new coordinate system, Eq. (S5) takes the following form

$$\begin{aligned}\frac{\partial f}{\partial \tau} =&\frac{\partial}{\partial \zeta}\left(D(n)\frac{\partial f}{\partial \zeta}\right) + \left[\frac{2D(n)}{n} + S(n)\right]\frac{dn}{d\zeta}\frac{\partial f}{\partial \zeta} + v_0\frac{\partial f}{\partial \zeta} \\ &+ D(n)\frac{\partial^2 f}{\partial \eta^2} - \left[A^{(1)} - A^{(2)}\right]\left[\frac{A^{(1)}}{A^{(1)} - A^{(2)}} - f\right]\frac{dn}{d\zeta}\frac{\partial f}{\partial \eta},\end{aligned} \tag{S15}$$

where we approximated $\frac{\partial \zeta}{\partial \tau}$ by v_0 to the first order in $A^{(1)} - A^{(2)}$ and assumed, as before, that n depends only on ζ.

To proceed, we assume a time-scale separation between the fast dynamics along ζ that quickly equilibrate the values of f across the thickness of the front and the slow dynamics along the η direction. The fast dynamics are given by the first three terms in Eq. (S15) and can be expressed as

$$\left(\frac{\partial f}{\partial \tau}\right)\bigg|_{\text{fast}} = L_\zeta f, \tag{S16}$$

where L_ζ is a linear operator that is given by

$$L_\zeta = \frac{\partial}{\partial \zeta}\left(D(n)\frac{\partial}{\partial \zeta}\right) + \left[\frac{2D(n)}{n} + S(n)\right]\frac{dn}{d\zeta}\frac{\partial}{\partial \zeta} + v_0\frac{\partial}{\partial \zeta}. \tag{S17}$$

It is clear that $f = \text{const}$ is an eigenfunction of L_ζ with a zero eigenvalue. Therefore, the solution of Eq. (S17) approaches $f = \text{const}$ in the long-time limit. The value of this constant can obtained by multiplying Eq. (S17) by $l_0(\zeta)$, the left eigenfunction of L_ζ with zero eigenvalue, and then integrating over ζ. After integrating by parts, the right hand side vanishes, so $\frac{\partial}{\partial \tau}\int_{-\infty}^{+\infty} l_0 f d\zeta = 0$, and the projection of f on l_0 is conserved. The value of this left eigenfunction can be easily obtained by the standard methods [3, 6] and is given by[2]

$$l_0 = n^2(\zeta)e^{\int_0^\zeta \frac{v_0 + S(n(\tilde{\zeta}))}{D(n(\tilde{\zeta}))}d\zeta}. \tag{S18}$$

We can now eliminate the fast dynamics by projecting both sides of Eq. (S15) on $l_0(\zeta)$. This procedure provides a natural definition of the coarse-grained variable $f(t, \eta)$ in terms of $f(t, \zeta, \eta)$

$$f(t, \eta) = \frac{\int_{-\infty}^{+\infty} l_0(\zeta) f(\tau, \zeta, \eta) d\zeta}{\int_{-\infty}^{+\infty} l_0(\zeta) d\zeta}, \tag{S19}$$

and leads to the following equation for $f(t, \eta)$

$$\frac{\partial}{\partial \tau}f(t, \eta) = D_f\frac{\partial^2}{\partial \eta^2}f(t, \eta) + \beta[f^* - f(t, \eta)]\frac{\partial}{\partial \eta}f(t, \eta), \tag{S20}$$

where

[2] This left eigenfunction is related to the probability that a mutant arises at position ζ and reaches fixation at the front some time afterwards [3, 6, 10].

$$D_f = \frac{\int_{-\infty}^{+\infty} l_0(\zeta) D(n(\zeta)) d\zeta}{\int_{-\infty}^{+\infty} l_0(\zeta) d\zeta}, \tag{S21}$$

$$\beta = -\frac{\int_{-\infty}^{+\infty} l_0(\zeta)[A^{(1)}(n(\zeta)) - A^{(2)}(n(\zeta))]\frac{dn}{d\zeta} d\zeta}{\int_{-\infty}^{+\infty} l_0(\zeta) d\zeta}, \tag{S22}$$

and

$$f^* = \frac{\int_{-\infty}^{+\infty} l_0(\zeta) A^{(1)}(n(\zeta))\frac{dn}{d\zeta} d\zeta}{\int_{-\infty}^{+\infty} l_0(\zeta)[A^{(1)}(n(\zeta)) - A^{(2)}(n(\zeta))]\frac{dn}{d\zeta} d\zeta}. \tag{S23}$$

Note that we assumed that $f(\tau, \zeta, \eta) \approx f(\tau, \eta)$ in all terms representing the slow dynamics along η.

The final step of the derivation is to change from τ and η back to t and x. To the first order in $\frac{\partial h}{\partial x}$, we find that $\frac{\partial f}{\partial \eta} = \frac{\partial f}{\partial x}$ and $\frac{\partial f}{\partial \tau} = \frac{\partial f}{\partial t} + \frac{\partial f}{\partial x}\frac{\partial x}{\partial \tau} = \frac{\partial f}{\partial t} + v_0 \frac{\partial f}{\partial x}\frac{\partial h}{\partial x}$. The last equality follows from the geometrical fact that a stationary point on a front tilted by a small angle $\frac{\partial h}{\partial x}$ moves with velocity $-v_0\frac{\partial h}{\partial x}$ with respect to the x-axis. Upon performing these substitutions, Eq. (S20) takes the following form

$$\frac{\partial f}{\partial t} = D_f \frac{\partial^2 f}{\partial x^2} + \beta(f^* - f)\frac{\partial f}{\partial x} + v_0 \frac{\partial h}{\partial x}\frac{\partial f}{\partial x}. \tag{S24}$$

Equation (S24) is the main result of this subsection. The first term of this equation describes the diffusive intermixing of the strains. The second term accounts for the movement of strains due to chirality. The third term describes the "kinematic" coupling between front shape and front composition and accounts for the sliding of a tilted front relative to the x-axis. In other words, the species fractions at a given x change simply because the same x corresponds to different locations on the front (specified by η) at different times. Without the last term, Eq. (S24) is the viscous Burgers' equation that describes dissipative flow of conserved quantities in one spatial dimension and is used as a toy model of fluid dynamics and traffic flow [11–14]. We find that the behavior of the boundaries is largely captured by the Burgers' equation except the last term in Eq. (S24) introduces an extra drift down the slopes of $h(t, x)$. The dynamics of the boundaries is discussed in a separate section below.

The primary results of this section are Equations (S24) and (S12) which couples the shape of the front with population dynamics occuring at the front. Such phenomenological models have been proposed to study systems such as the surface growth of binary alloy films [9]

S3 Derivation of the effective theory from phenomenological considerations

This section derives the effective theory stated by Eqs. (S12) and (S24) from phenomenological considerations. Specifically, we perform a gradient expansion assuming that the spatial variations are slow and consider the most general set of equations for $h(t, x)$ and $f(t, x)$ that contain terms with up to two spatial derivatives:

$$\frac{\partial h}{\partial t} = H_0 + H_{h_x}\frac{\partial h}{\partial x} + H_{f_x}\frac{\partial f}{\partial x} + H_{h_{xx}}\frac{\partial^2 h}{\partial x^2} + H_{(h_x)^2}\left(\frac{\partial h}{\partial x}\right)^2 + H_{h_x f_x}\frac{\partial h}{\partial x}\frac{\partial f}{\partial x} + H_{(f_x)^2}\left(\frac{\partial f}{\partial x}\right)^2 + H_{f_{xx}}\frac{\partial^2 f}{\partial x^2}, \quad \text{(S25)}$$

and

$$\frac{\partial f}{\partial t} = F_0 + F_{h_x}\frac{\partial h}{\partial x} + F_{f_x}\frac{\partial f}{\partial x} + F_{h_{xx}}\frac{\partial^2 h}{\partial x^2} + F_{(h_x)^2}\left(\frac{\partial h}{\partial x}\right)^2 + F_{h_x f_x}\frac{\partial h}{\partial x}\frac{\partial f}{\partial x} + F_{(f_x)^2}\left(\frac{\partial f}{\partial x}\right)^2 + F_{f_{xx}}\frac{\partial^2 f}{\partial x^2}, \quad \text{(S26)}$$

where every coefficient could depend on f, but not on h because the space is invariant under translations.

To understand the role and origin of each term, it is important to know the effects of the following transformations: reflection in the y-axis ($x \to -x$), exchange of strains labels, and rotation of the front by a small angle ϕ. Let us examine each transformation in more detail. Reflection in the y-axis changes the sign of x and the signs of the chiralities of the strains. By chiralities, we do not necessarily mean the ones defined in Eq. (S2), but any two numbers that capture the chiral nature of the two strains such that the chirality is zero for non-chiral strains, positive for left-moving strains, and negative for right-moving strains. All terms that appear in Eqs. (S25) and (S26) due to chirality can be expanded in Taylor series in powers of the difference between the chiralities of the two strains $\Delta A = A^{(1)} - A^{(2)}$. The constant term in the series must vanish because the morphology of a compact microbial colony is the same for any two strains with equal chiralities regardless of the magnitude of this chirality. In addition to ΔA, the terms could depend on $f^* = A^{(1)}/(A^{(1)} - A^{(2)})$, which does not change when both $A^{(1)}$ and $A^{(2)}$ change sign. These considerations and the fact that reflection in the y should not alter the nature of the competition leads us to the following conclusion: Eqs. (S25) and (S26) must remain invariant under $x \to -x$ and $\Delta A \to -\Delta A$.

The model formulation should also be invariant under the exchange of species labels. This exchange results in the following transformations $f \to 1-f$, $\Delta A \to -\Delta A$, and $f^* \to 1 - f^*$, which impose another symmetry requirement on Eqs. (S25) and (S26).

Rotation by a small angle ϕ leads to a change of variable from x and h to $\tilde{x} = x/\cos\phi$ and $\tilde{h} = h\cos\phi - x\sin\phi$. When space is isotropic this transformation should also leave Eqs. (S25) and (S26) unchanged. However, because these equations assume that $\frac{\partial h}{\partial x}$ is small, we only require invariance up to the second order in ϕ. Note that, this symmetry does not hold for non-isotropic spaces, which appear, for example, in lattice-based simulations. In the equation for $f(t,x)$ a rotation by angle ϕ also modifies $\frac{\partial f}{\partial t}$ to $\frac{\partial f}{\partial t} - v_0 \frac{\partial f}{\partial \tilde{x}}\tan\phi$.

In addition to the above symmetries, we must also require that strains cannot be spontaneously created. This requirement implies that all of the terms in Eq. (S24) should vanish when $f \to 0$ and $f \to 1$.

We now analyze each of the terms in Eqs. (S25) and (S26) separately and then discuss the implications of these results for the form of the effective model of the competition at the front.

Term $H_0 + H_{(h_x)^2}\left(\frac{\partial h}{\partial x}\right)^2$. These two terms are considered together because rotational symmetry requires that $H_{(h_x)^2} = H_0/2$. When f and h do not depend on x, H_0 is the only term contributing to $\frac{\partial h}{\partial t}$, so we can interpret it as the front velocity v_0. This velocity could depend on f, for example, when species are cross-feeding or one of the species is more fit. Even when chirality is the only difference between the species, v_0 could still be f-dependent, but this dependence should scale as $(\Delta A)^2$ because the preceding term in the Taylor expansion must vanish due to mirror symmetry[3]. Thus, $v_0 = \text{const}$ is a reasonable approximation

[3]If the two strains have identical expansion velocities when grown by themselves, then the correction to v_0 should scale as $f(1-f)(\Delta A)^2$ to the leading order in ΔA.

for small differences in strain chiralities. We also note that in lattice-based models, which explicitly violate rotational symmetry, $H_{(h_x)^2} = H_0/2$ does not have to hold. The deviations from this equality indicate how the expansion velocity depends on the orientation of the front relative to the lattice.

Term $H_{h_{xx}}\frac{\partial^2 h}{\partial x^2}$. For $H_{h_{xx}}$ = const, this term corresponds to the diffusion term in Eq. (S12). The dependence on f is possible, but should scale as $(\Delta A)^2$ for the same reasons as above. The three terms discussed so far constitute the standard KPZ equation without noise [8].

Term $H_{f_x}\frac{\partial f}{\partial x}$. This term corresponds to $\alpha\frac{\partial f}{\partial x}$ in Eq. (S12) and is the only new term that appeared due to chirality in our derivation of the effective model via the moving-boundary approximation. Because $\frac{\partial f}{\partial x}$ changes sign under mirror symmetry, H_{f_x} should be odd in ΔA. Our expression for α in Eq. (S14) can therefore be viewed as the first term in the Taylor expansion of H_{f_x} in powers of ΔA. The symmetry under the exchange of the labels further implies that α can not depend on f linearly, so our result that α = const should be a reasonable approximation to a more general model. In the reaction-diffuson model, the dependence of α on f can arise from the dependence of $A^{(\alpha)}$ on f.

Term $H_{h_x}\frac{\partial h}{\partial x}$. This term violates rotational symmetry and, therefore, must be absent in a continuum model, but it should appear in a lattice-bades model. Because $\frac{\partial h}{\partial x}$ changes sign under the mirror symmetry, $H_{h_x} \sim \Delta A$ at the leading order. The label exchange symmetry imposes a further constraint[4] $H_{h_x} \approx \gamma(f^*_\gamma - f)$, where $\gamma \sim \Delta A$. Indeed, H_{h_x} simply equal to γ without the f-dependence changes sign under the label exchange and thus does not leave the equation invariant.

Term $H_{h_x f_x}\frac{\partial h}{\partial x}\frac{\partial f}{\partial x}$. Similar to the term just discussed, this term violates the rotational symmetry and, therefore, should be excluded. In lattice-based models, $H_{h_x f_x}\frac{\partial h}{\partial x}\frac{\partial f}{\partial x}$ is allowed, but we expect that its contribution is subleading to that from $H_{h_x}\frac{\partial h}{\partial x}$ because of the extra spatial derivative, which is small under our assumption of slow spatial variation. The mirror symmetry requires that $H_{h_x f_x} \sim (\Delta A)^2$.

Term $H_{(f_x)^2}\left(\frac{\partial f}{\partial x}\right)^2$. Since $\left(\frac{\partial f}{\partial x}\right)^2$ is invariant under $x \to -x$, this term can describe competition between non-chiral strains. One possible origin of this term in the reaction-diffusion model is the dependence of $D^{(1)} - D^{(2)}$ on f. When $H_{(f_x)^2}\left(\frac{\partial f}{\partial x}\right)^2$ arises due to chirality differences, we expect that $H_{(f_x)^2} \sim (\Delta A)^2$ due to the mirror symmetry.

Term $H_{f_{xx}}\frac{\partial^2 f}{\partial x^2}$. Similar to the term just discussed, $H_{f_{xx}}\frac{\partial^2 f}{\partial x^2}$ could appear in a model without any chirality difference, for example, due to unequal dispersal coefficients of the two strains ($D^{(1)} \neq D^{(2)}$). The reflection symmetry and the symmetry due to the exchange of labels suggest that $H_{f_{xx}}$ should scale as $\Delta A(f^* - f)$ to the lowest order in ΔA assuming the simplest dependence on f. More complex dependence is also possible, for example, $F_{h_x} = \Delta A(f^* - f)Q(f(1 - f))$, where Q is an arbitrary function.

This completes the analysis of the terms in Eq. (S25), so we proceed with the same analysis for Eq. (S26).

[4]In the simplest case, the relative chirality could be quantified by a single f^* entering all relevant terms. However, the possible dependence of $A^{(\alpha)}$ on n together with Eq. (S23) suggest that slightly different f^* might appear in different terms. To account for such a possibility, we added an index to f^* that signifies the term in the equation that this particular f^* is associated with. For simplicity, we omit such indexes in the rest of this section.

Term F_0 . This term arises due to the difference in the growth rates of the strains, $g^{(\alpha)}$, and describes the effects of natural selection such as exclusion, coexistence, or bistability. Because we are interested in the effects of chirality, we only consider strains with the same fitness, for which $F_0 = 0$.

Term $F_{h_x}\frac{\partial h}{\partial x}$. Since this term violates rotational symmetry, it requires that the space is not isotropic, for example, due to using a lattice-based model to simulate population dynamics. The mirror symmetry requires that F_{h_x} is proportional to ΔA at the lowest order in the difference between the chiralities. Further, F_{h_x} must vanish when $f = 0$ or $f = 1$ because the strains cannot interconvert between each other. Thus, the simplest form of this term is $F_{h_x} \sim \Delta A f(1-f)$, but a more complicated dependence on f is possible similar to the case of $H_{f_{xx}}\frac{\partial^2 f}{\partial x^2}$ discussed above. One potentially important effect due to F_{h_x} is the induced selection on the sides of the bulges formed around in-flow boundaries. When $F_{h_x} > 0$, selection favors the invasion of either side of the bulge by the strain dominating the other side. Hence, positive F_{h_x} promotes strain intermixing. Negative F_{h_x} opposes strain intermixing because selection suppresses the growth of the strain dominating the opposite side of the bulge.

Term $F_{f_x}\frac{\partial f}{\partial x}$. This term is equivalent to $\beta(f^* - f)\frac{\partial f}{\partial x}$ in Eq. (S24). To satisfy the requirements of the mirror and label exchange symmetries, F_{f_x} should scale as $\Delta A(f^* - f)$ consistent with the results from the reaction-diffusion model. More general dependence on f is also possible; for example F_{f_x} could scale as $\Delta A(f^* - f)Q(f(1-f))$, where Q is an arbitrary function. Thus, β could depend on f in Eq. (S24). This dependence, however, is unlikely to alter any of the qualitative conclusions because the main aspects of the strain motion due to chirality are already captured by constant β. In particular, the dependence of β on f contributes only to the width of the strain boundaries and is irrelevant in the domains occupied by a single strain, where $f = \text{const}$. At the boundaries, however, the phenomenological model does not account for other important factors such as stochasticity and large spatial gradients, so neglecting the dependence of β on f is a reasonable simplification.

Term $F_{h_{xx}}\frac{\partial^2 h}{\partial x^2}$. Since $\frac{\partial^2 h}{\partial x^2}$ is invariant under $x \to -x$, this term could occur even without chirality differences. When it occurs due to chirality differences, the symmetries require that $F_{h_{xx}} \sim (\Delta A)^2(f^* - f)$. More importantly, a term without a gradient of f cannot arise in our reaction-diffusion model because $f = \text{const}$ is a solution of Eq. (S5) for any $h(t, x)$. Therefore, the origin of $F_{h_{xx}}\frac{\partial^2 h}{\partial x^2}$ must be related to the difference in a fitness component of strains that couples the relative growth rate and the local front curvature.

Term $F_{(h_x)^2}\left(\frac{\partial h}{\partial x}\right)^2$. All of the comments that we made about $F_{h_{xx}}\frac{\partial^2 h}{\partial x^2}$ apply to this term as well. In addition, $\left(\frac{\partial h}{\partial x}\right)^2$ is not invariant under rotations, so this term can arise only when rotational symmetry is broken, for example, due to lattice effects.

Term $F_{h_x f_x}\frac{\partial h}{\partial x}\frac{\partial f}{\partial x}$. To satisfy rotational invariance, we must set $F_{h_x f_x}$ to v_0. Lattice effects however can result in a violation of this constraint and make the boundary between two strains move with different velocities (relative to the front) for different front orientations with respect to the lattice.

Term $F_{(f_x)^2}\left(\frac{\partial f}{\partial x}\right)^2$. Since $\frac{\partial^2 h}{\partial x^2}$ is invariant under $x \to -x$, this term could occur even without chirality differences. When it occurs due to chirality differences, the mirror and label symmetries require that $F_{(f_x)^2} \sim (\Delta A)^2(f^* - f)$, i.e. this term is of higher order in the difference between the chiralities. In addition, this term violates another symmetry: Galilean invariance. Because the term is independent of h, we can consider a flat front containing left and right biased strains. Since one can always choose a reference frame moving along the front in which the biases are equal and opposite, $F_{(f_x)^2}$ cannot depend on f^*, which is not invariant

under this transformation. The presence of f^* is however required by the symmetry under the exchange of the labels. Therefore, $F_{(f_x)^2}\left(\frac{\partial f}{\partial x}\right)^2$ can appear only in models that contain a preferred reference frame and thereby explicitly break Galilean invariance. This situation arises naturally in lattice-based models because their formulation often depends on the lattice being at rest.

Term $F_{f_{xx}}\frac{\partial^2 f}{\partial x^2}$. This term corresponds to $D_f\frac{\partial^2 f}{\partial x^2}$ in Eq. (S24), which describes the diffusive intermixing of the strains. The diffusion coefficient could in principle depend on f, but the mirror symmetry requires that this dependence is at least quadratic in ΔA. Therefore, in the following, we assume that D_f = const consistent with the results from the reaction-diffusion model (Eq. S21).

After analyzing all of the terms in Eqs. (S25) and (S26), we conclude that, to the leading order in ΔA, Eqs. (S12) and (S24) capture all possible contributions to the evolution of $h(t,x)$ and $f(t,x)$ consistent with the symmetries of the problem. Therefore, Eqs. (S12) and (S24) should hold even when some of the assumptions underlying their derivation from the reaction-diffusion model are violated. The phenomenological description further provides a clear path to accounting for the lattice effects in simulations and to including the effects that higher order in ΔA.

S4 Deterministic behavior of in-flow and out-flow boundaries in flat fronts

This section considers the dynamics of isolated domain boundaries located in flat regions of the front without tilt ($\frac{\partial h}{\partial x} = 0$). We determine the velocity of in-flow and out-flow boundaries and explain why these two types of boundaries have different width. Throughout this section we neglect fluctuations in f due to genetic drift. Demographic fluctuations, however, play a major role in our simulations and microbial experiments [15, 16], so the discussion of boundary shape is included largely to provide intuitive understanding rather than quantitative description of the dynamics. In the limit of large carrying capacity, N, our simulations do reproduce the behavior described below, but for moderate N genetic drift leads to a qualitative change in the dynamics. Specifically, strong demographic fluctuations limit the spreading of domain boundaries and restrict strain intermixing to a finite region [17]. The deterministic dynamics discussed below influence the size of this region and make in-flow boundaries wider than out-flow boundaries.

Boundary velocity

By setting $\frac{\partial h}{\partial x}$ to zero in Eq. (S24), we obtain

$$\frac{\partial f}{\partial t} = D_f\frac{\partial^2 f}{\partial x^2} + \beta(f^* - f)\frac{\partial f}{\partial x}, \tag{S27}$$

which becomes equivalent to the viscous Burgers' equation upon identifying $f - f^*$ with the flow velocity [13, 14]. The Burgers' equation has been extensively studied, so the properties of its solutions are well-characterized [12–14]. The main purpose of this section is to provide a short summary of the standard results in the language of our model.

The analysis of Eq. (S27) simplifies upon changing into a reference frame moving with velocity

$$v_{\|} = (\frac{1}{2} - f^*)\beta. \tag{S28}$$

Indeed, by letting $\mathfrak{z} = x - v_{\|}t$, we find that Eq. (S27) takes the following form

$$\frac{\partial f}{\partial t} = D_f \frac{\partial^2 f}{\partial \mathfrak{z}^2} + \beta \left(\frac{1}{2} - f \right) \frac{\partial f}{\partial \mathfrak{z}}, \tag{S29}$$

which is the same as Eq. (S27) but with f^* replaced by $\frac{1}{2}$. Because $f^* = \frac{1}{2}$ corresponds to exactly opposite chiralities (see Eq. (S23)), the dynamics in the reference frame moving with velocity $v_{\|}$ are equivalent to those of two oppositely chiral strains. From the symmetry considerations, it follows that the boundaries between the two oppositely chiral strains must remain stationary. Therefore, $v_{\|}$ defined by Eq. (S28) is the velocity of the boundaries in the original reference frame.[5]

Equation (S28) can be obtained more directly. The first step is to observe that Eq. (S27) conserves the integral of f over dx because $-\frac{\partial f}{\partial t}$ equals the divergence of the flux $J = -D_f \frac{\partial f}{\partial x} + \frac{\beta}{2}(f - f^*)^2$. The second step is to find a reference frame where the fluxes in and out of the domain boundary balance. Due to the conservation of $\int f dx$, the boundary must remain stationary when there is no net flux, so the boundary velocity is given by the velocity of the reference frame. This approach yields the same value of $v_{\|}$ as in Eq. (S28).

Shape of boundaries

We now consider the shapes of in-flow and out-flow boundaries in the co-moving reference frame. For an out-flow boundary, Eq. (S29) admits the following stationary solution

$$f(\mathfrak{z}) = \frac{1}{2} \left[1 - \tanh \left(\frac{\beta}{4D_f} \mathfrak{z} \right) \right]. \tag{S30}$$

Thus, out-flow boundaries reach a finite width on the order of D_f/β even in the absense of noise. The out-flouw boundaries become infinitely sharp in the limit of $D_f \to 0$, which corresponds to the inviscid Burgers' equation. Such sharp changes in f are termed shocks in the language of nonlinear partical differential equations.

The behavior of in-flow boundaries is completely different. Instead of reaching a fixed shape, they widen indefinitely. The temporal evolution of the boundary shape can be determined by solving Eq. (S29) equation exactly. This is done by the following Cole-Hopf transformation:

$$f = \frac{1}{2} + \frac{2D_f}{\beta} \frac{\partial \ln w}{\partial \mathfrak{z}}, \tag{S31}$$

which yields the standard diffusion equation for w:

$$\frac{\partial w}{\partial t} = D_f \frac{\partial^2 w}{\partial \mathfrak{z}^2}. \tag{S32}$$

[5] This result assumes that f is either 0 or 1 on both sides of the boundary, as it is in our simulations. The more general result is that $v_{\|} = \beta \left(\frac{f_+ + f_-}{2} - f^* \right)$, where f_- and f_+ are the values of f on the two sides of the boundary.

The qualitative behavior of in-flow boundaries can also be obtained in a simpler way by neglecting the effect of diffusion; this is a very good approximation on intermediate time scales [14]. Setting D_f to zero reduces Eq. (S27) to a first order partial differential equation, which can be solved by the method of characteristics. For a step-function initial condition ($f(0,x) = \theta(x)$), the solution reads

$$f(t,x) = \begin{cases} 1 \;\; x > \beta(1-f^*)t, \\ f^* + \dfrac{x}{\beta t} \;\; x \in [-\beta f^* t,\ \beta(1-f^*)t], \\ 0 \;\; x < -\beta f^* t. \end{cases} \tag{S33}$$

Thus, the width of the boundary grows as βt. While this widening is arrested by strong genetic drift, we nevertheless expect in-flow boundaries to be wider than out-flow boundaries. Our simulations agree with this expectation; see Figs.2 and 3 in the main text.

Interaction between boundaries in the Burgers' equation

So far, we considered the dynamics of isolated boundaries, which is a good description for times shorter than the time required for a widening in-flow boundary to reach the nearest out-flow boundary. Beyond this time scale, the boundaries begin to interact. This interaction is largely irrelevant in the context of microbial colonies because strong genetic drift arrests the widening of out-flow boundaries; as a result, the boundaries come in contact only via a random walk or due to the bulge-induced motion.

For completeness, we briefly destribe the effect of boundary interactions in the context of the Burgers' equation, but we emphasize that these dynamics do not occur in the context of competition between chiral strains in growing colonies. Burgers' equation predicts that boundary interaction induces the motion of out-flow boundaries in the opposite direction compared to results of our simulations and the prediction based on the bulge-induced motion of out-flow boundaries. The interaction between boundaries also reduces spatial variations in $f(t,x)$ until the stationary solution $f(t,x) = \text{const}$ is reached. The deviations from $f(t,x) = \text{const}$ decay as $t^{-1/2}$ for a pair of isolated boundaries and as t^{-1} for a periodic array of boundaries [14].

S5 Deterministic solution for bulge and dip shape near a sharp boundary

In this section, we obtain the shape of bulges and dips at in-flow and out-flow boundaries in the effective theory given by Eqs. (S12) and (S24). In general, this system of coupled equations is difficult to solve, so we resort to two additional approximations. The first approximation is that we decouple the equations and solve for $h(t,x)$ by assuming that $f(t,x)$ is known. The second approximation is that we consider the limit of a very sharp boundary and assume that $f(t,x) = \theta(\pm(x - v_{\|}t))$. Here, + refers to in-flow and − to out-flow boundaries respectively; $\theta(x)$ is the Heaviside step function; and $v_{\|}$ is the velocity of the boundary. This assumption is justified in the limit of strong genetic drift, which prevents strain intermixing through local stochastic exctinctions of one of the strains. In microbial colonies, genetic drift is typically very strong and one does indeed observe very narrow boundaries [15, 16, 18]. The sharp boundary approximation can also be viewed as a simplifying assumption that preserves the qualitative aspects of the problem by neglecting the effects due to an additional length scale associated with boundary width.

The velocity of the boundary depends on f^*. To the first approximation in ΔA, it is natural to neglect the contribution of the front shape near the boundary and assume that $v_{\|}$ is given by its value for a flat front in Eq. (S28). This assumption, however, needs a careful consideration because we show in this and following

sections that the tilt of the front due to a bulge is proportional to ΔA and that this tilt induces a velocity linear in ΔA for a boundary trapped on the slope of the bulge. Because a boundary associated with a bulge or a dip is positioned between two slopes tilted in opposite directions, the induced velocities could effectively cancel[6] at least to the first order in ΔA. For a special case of $f^* = \frac{1}{2}$, one can however argue that $v_\parallel = 0$ purely from the symmetry considerations. Below, we analyze this simpler case first and then generalize the result for $v_\parallel \neq 0$. Our results in this section do not depend on the specific expression for the boundary velocity, so we treat $v_\parallel$ as an arbitrary parameter to preserve generality.

Bulge shape at a stationary in-flow boundary, $f^* = \frac{1}{2}$

Upon substituting $f(t, x) = \theta(x)$ into Eq. (S12), we obtain

$$\frac{\partial h}{\partial t} = v_0 + \frac{v_0}{2}\left(\frac{\partial h}{\partial x}\right)^2 + D_h \frac{\partial^2 h}{\partial x^2} + \alpha\delta(x), \tag{S34}$$

where $\delta(x)$ is the Dirac delta function. A reasonable choice of the initial conditions is $h(0, x) = 0$.

Before describing the formal solution, let us observe that the long-time limit of $h(t, x)$ can be immediately guessed from Eq. (S34). Indeed, the first term contributes an additive term to the solution equal to $v_0 t$. The Delta function leads a discontinuity in $\frac{\partial h}{\partial x}$ at $x = 0$ and thereby induces front tilt, which increases the front velocity from v_0 to a higher value through $\frac{v_0}{2}\left(\frac{\partial h}{\partial x}\right)^2$ term. The diffusion term is exactly zero provided $h(t, x)$ is a linear function of x. Thus, the solution should be a linear combination of a term proportional to time t and a term proportional to the absolute value of the position $|x|$. Direct substitution of this ansatz reveals that

$$h(t, x)_{\text{bulge}} = v_0\left(1 + \frac{\alpha^2}{8D_h^2}\right)t - \frac{\alpha}{2D_h}|x|. \tag{S35}$$

We now proceed to derive this result more formally by solving Eq. (S34) for all t. The first step is based on the Cole-Hopf transformation [14]:

$$h = v_0 t + \frac{2D_h}{v_0}\ln u, \tag{S36}$$

which leads to a linear equation for the new dynamical variable u

$$\frac{\partial u}{\partial t} = D_h \frac{\partial^2 u}{\partial x^2} + \frac{v_0 \alpha}{2D_h} u\delta(x). \tag{S37}$$

The initial condition transforms to $u(0, x) = 1$. We note in passing that, for $\alpha < 0$, Eq. (S37) coincides with the equation that describes the decay of neutral diversity in a one-dimensional population (Eq. (33) in Ref. [15]); therefore, our solution (Eq. S44) provides a closed-form expression for the decay of heterozygosity.

To proceed, we replace the term with the delta function by the following boundary conditions

[6] One needs to analyze the deviations from the linear shape of the bulge to make this argument precise. In particular, no modification of $v_\parallel$ is expected if the boundary is located on the flat portion of the bulge's top where $\frac{\partial h}{\partial x} = 0$; see Eq. (S59).

$$
\begin{aligned}
\lim_{x\to+0}\frac{\partial u}{\partial x} &= -\frac{v_0\alpha}{4D_h^2}u(0),\\
\lim_{x\to-0}\frac{\partial u}{\partial x} &= \frac{v_0\alpha}{4D_h^2}u(0),
\end{aligned}
\tag{S38}
$$

which are obtained by integrating Eq. (S37) over x in the vicinity of $x = 0$, neglecting the contribution form $\frac{\partial u}{\partial t}$, and using the mirror symmetry of the problem. After this step, we apply the Laplace transform in time and obtain the solution of Eq. (S37)

$$
u(s,x) = \frac{1}{s}\left(1 + \frac{\alpha v_0}{4D_h^2}\frac{e^{-\sqrt{\frac{s}{D_h}}|x|}}{\sqrt{\frac{s}{D_h}} - \frac{\alpha v_0}{4D_h^2}}\right), \tag{S39}
$$

in terms of $u(s,x) = \int_0^{+\infty} e^{st}u(t,x)dt$.

The inverse Laplace transform then provides the solution for $u(t,x)$:

$$
u(t,x) = \frac{1}{2\pi i}\int_{a-i\infty}^{a+i\infty}\frac{e^{st}}{s}\left(1 + \frac{\alpha v_0}{4D_h^2}\frac{e^{-\sqrt{\frac{s}{D_h}}|x|}}{\sqrt{\frac{s}{D_h}} - \frac{\alpha v_0}{4D_h^2}}\right)ds, \tag{S40}
$$

where a is a positive number larger than the real part of any singularity of the integrand.

The integrand in Eq. S40 has a branch cut along the negative x-axis and two poles at $s = 0$ and $s = \frac{\alpha^2 v_0^2}{16D_h^3}$. Therefore, we can simplify the integral as follows

$$
u(t,x) = \theta(t)\left[1 + 2e^{\frac{v_0}{2D_h}\left(\frac{v_0\alpha^2}{8D_h^2}t - \frac{\alpha}{2D_h}|x|\right)} + \frac{1}{2\pi i}\frac{\alpha v_0}{4D_h^2}\int_{\mathcal{C}}\frac{e^{st}}{s}\frac{e^{-\sqrt{\frac{s}{D_h}}|x|}}{\sqrt{\frac{s}{D_h}} - \frac{\alpha v_0}{4D_h^2}}ds\right], \tag{S41}
$$

where the contour $\mathcal{C}$ first goes just under the negative x-axis from $-\infty - 0i$ to 0, then around $s = 0$, and finally just above the negative x-axis from 0 to $\infty + 0i$. In the following, we consider only $t > 0$ and, therefore, omit $\theta(t)$ in all formulas.

It is easy to see that the second term in Eq. (S41) describes the long-time limit of $u(t,x)$. Indeed, this term arises due to the pole at $s = \frac{\alpha^2 v_0^2}{16D_h^3}$, which is the dominant singularity because it has the largest real part. The first term in Eq. (S41) arises due to the initial conditions and the final term describes transient dynamics and the transition from the asymptotic long-time limit at small x to unperturbed front, $u = 1$, at large x.

The integral in Eq. (S41), which we denote as I, can be evaluated exactly. To carry out this calculation, it is convenient to evaluate $\frac{\partial I}{\partial t}$ first because this differentation removes the pole at $s = 0$. Then, we combine the integrals above and below the negative x-axis and remove the radicals by introducing $p = \sqrt{s}$. The result reads

$$
\frac{\partial I}{\partial t} = \frac{\alpha v_0}{2\pi D_h\sqrt{D_h t}}\int_0^{+\infty}\frac{p^2\cos\frac{p|x|}{\sqrt{D_h t}} - \frac{\alpha v_0 p}{4D_h}\sqrt{\frac{t}{D_h}}\sin\frac{p|x|}{\sqrt{D_h t}}}{p^2 + \frac{\alpha^2 v_0^2}{16D_h^3}t}e^{-p^2}dp. \tag{S42}
$$

This integral can be evaluated directly using the following formulas from Ref. [19]:

$$\int_0^{+\infty} e^{-bx^2}\cos(ax)dx = \frac{1}{2}\sqrt{\frac{\pi}{b}}e^{-\frac{a^2}{4b}},$$
$$\int_0^{+\infty} e^{-bx^2}\sin(ax)\frac{xdx}{x^2+\gamma^2} = -\frac{\pi}{4}e^{b\gamma^2}\left[2\sinh(a\gamma)+e^{-a\gamma}\operatorname{erf}\left(\gamma\sqrt{b}-\frac{a}{2\sqrt{b}}\right)-e^{a\gamma}\operatorname{erf}\left(\gamma\sqrt{b}+\frac{a}{2\sqrt{b}}\right)\right],$$
$$\int_0^{+\infty} e^{-bx^2}\cos(ax)\frac{dx}{x^2+\gamma^2} = \frac{\pi}{4\gamma}e^{b\gamma^2}\left[2\cosh(a\gamma)-e^{-a\gamma}\operatorname{erf}\left(\gamma\sqrt{b}-\frac{a}{2\sqrt{b}}\right)-e^{a\gamma}\operatorname{erf}\left(\gamma\sqrt{b}+\frac{a}{2\sqrt{b}}\right)\right]. \tag{S43}$$

where erf(x) is the error function [19].

To obtain I, we integrate $\frac{\partial I}{\partial t}$ over t; I at $t=0$ is determined from Eq. (S41) and the fact that $u(0,x)=1$. After judicious integration by parts, we find that

$$u(t,x) = e^{\frac{v_0^2\alpha^2}{16D_h^3}t-\frac{v_0\alpha}{4D_h^2}|x|}\operatorname{erfc}\left(\frac{|x|}{2\sqrt{D_ht}}-\frac{\alpha v_0}{4D_h}\sqrt{\frac{t}{D_h}}\right)+\operatorname{erf}\frac{|x|}{2\sqrt{D_ht}}, \tag{S44}$$

where erfc(x) is the complementary error function [19]. The solution for $h(t,x)$ is obtained by changing variables back from u to h:

$$h(t,x)_{\text{bulge}} = v_0\left(1+\frac{\alpha^2}{8D_h^2}\right)t-\frac{\alpha}{2D_h}|x|+\frac{2D_h}{v_0}\ln\left[\operatorname{erfc}\left(\frac{|x|}{2\sqrt{D_ht}}-\frac{\alpha v_0}{4D_h}\sqrt{\frac{t}{D_h}}\right)+e^{-\frac{v_0^2\alpha^2}{16D_h^3}t+\frac{v_0\alpha}{4D_h^2}|x|}\operatorname{erf}\left(\frac{|x|}{2\sqrt{D_ht}}\right)\right]. \tag{S45}$$

For a fixed x and large t, the last term in Eq. (S45) approaches a constant, so the long-time limit is given by the first two terms, which specify the height and shape of the bulge.

Dip shape at a stationary out-flow boundary, $f^* = \frac{1}{2}$

The analysis of the dip shape is essentially the same as above. Indeed, the substitution of $f(t,x)=\theta(-x)$ into Eq. (S12) yields

$$\frac{\partial h}{\partial t} = v_0 + \frac{v_0}{2}\left(\frac{\partial h}{\partial x}\right)^2 + D_h\frac{\partial^2 h}{\partial x^2} - \alpha\delta(x), \tag{S46}$$

which is identical to Eq. (S34), but with the opposite sign in front of α. Therefore, the solution of Eq. (S46) is obtained by replacing α by $-\alpha$ in Eq. (S45):

$$h(t,x)_{\text{dip}} = v_0t + \frac{2D_h}{v_0}\ln\left[e^{\frac{v_0^2\alpha^2}{16D_h^3}t+\frac{v_0\alpha}{4D_h^2}|x|}\operatorname{erfc}\left(\frac{|x|}{2\sqrt{D_ht}}+\frac{\alpha v_0}{4D_h}\sqrt{\frac{t}{D_h}}\right)+\operatorname{erf}\frac{|x|}{2\sqrt{D_ht}}\right]. \tag{S47}$$

Note that we stated this result in a slightly different from compared to Eq. (S45) because the leading behavior in the long-time limit is now given by different terms; instead of approaching a constant, the complementary

error function now tends to zero and nearly cancels the exponential term in front of it. For large t and finite x, Eq. (S47) can be approximated by

$$h(t,x)_{\rm dip} \approx v_0 t + \frac{2D_h}{v_0}\ln\left(\frac{\frac{8D_h^2}{\sqrt{\pi}\alpha v_0}+|x|}{2\sqrt{D_h t}}\right). \tag{S48}$$

Thus, the shape of a dip is a curvilinear angle made by two logarithmic curves rather than a regular angle made by two straight lines as we found in the case of a bulge; see Fig.4 in the main text. The depth of the dip, $v_0 t - h(t,0)$, increases logarithmically in time as $\frac{D_h}{v_0}\ln\left(\frac{\pi\alpha^2 v_0^2 t}{16D_h^3}\right)$ and the width of the dip increases as $2\sqrt{D_h t}$. Because both the depth and the width grow much slower for a dip than for a bulge, we expect that the front shape is largely dominated by the locations of the bulges. Consistent with this expectation, our simulation revealed only modest dips that do not extend appreciably beyond the width of the corresponding out-flow boundaries. This observation holds regardless of the value of f^*.

The shape of a moving bulge, $f^* \neq \frac{1}{2}$

We now relax the assumption that $f^* = \frac{1}{2}$ and derive the asymptotic shape of a moving bulge. The calculation largely proceeds through the same steps as before starting with the following equation for $h(t,x)$:

$$\frac{\partial h}{\partial t} = v_0 + \frac{v_0}{2}\left(\frac{\partial h}{\partial x}\right)^2 + D_h\frac{\partial^2 h}{\partial x^2} + \alpha\delta(x - v_\parallel t). \tag{S49}$$

The change of variables from h to u according Eq. (S36) results in

$$\frac{\partial u}{\partial t} = D_h\frac{\partial^2 u}{\partial x^2} + \frac{v_0\alpha}{2D_h}u\delta(x - v_\parallel t). \tag{S50}$$

The next step is to shift into a reference frame moving with velocity $v_\parallel$ by defining a new spatial variable $\mathfrak{z} = x - v_\parallel t$. In terms of $\mathfrak{z}$, the equation for u takes the following form

$$\frac{\partial u}{\partial t} = D_h\frac{\partial^2 u}{\partial \mathfrak{z}^2} + v_\parallel\frac{\partial u}{\partial \mathfrak{z}} + \frac{v_0\alpha}{2D_h}u\delta(\mathfrak{z}). \tag{S51}$$

We proceed by performing the Laplace transform in time, which yields

$$D_h\frac{\partial^2 u}{\partial \mathfrak{z}^2} + v_\parallel\frac{\partial u}{\partial \mathfrak{z}} - su + \frac{v_0\alpha}{2D_h}u\delta(\mathfrak{z}) = -1, \tag{S52}$$

where we used the initial condition $u(0,x) = 1$. This linear equation can be solved for $\mathfrak{z} > 0$ and $\mathfrak{z} < 0$ separately under the constraints that $\lim_{\mathfrak{z}\to-\infty} u = \lim_{\mathfrak{z}\to+\infty} u = 0$. These two solutions are then matched by imposing continuity at $\mathfrak{z} = 0$ and the following condition due to the term with the delta function

$$\lim_{\mathfrak{z}\to+0}\frac{\partial u}{\partial \mathfrak{z}} - \lim_{\mathfrak{z}\to-0}\frac{\partial u}{\partial \mathfrak{z}} = -\frac{v_0\alpha}{2D_h^2}\lim_{\mathfrak{z}\to 0} u. \tag{S53}$$

The result reads

$$u = \frac{1}{s}\left[1 + \frac{\frac{\alpha v_0}{4D_h^2}}{\sqrt{\frac{s}{D_h} + \frac{v_\parallel^2}{4D_h^2}} - \frac{\alpha v_0}{4D_h^2}}\left(\theta(\mathfrak{z})e^{-\left(\frac{v_\parallel}{2D_h}+\sqrt{\frac{s}{D_h}+\frac{v_\parallel^2}{4D_h^2}}\right)\mathfrak{z}} + \theta(-\mathfrak{z})e^{-\left(\frac{v_\parallel}{2D_h}-\sqrt{\frac{s}{D_h}+\frac{v_\parallel^2}{4D_h^2}}\right)\mathfrak{z}}\right)\right]. \tag{S54}$$

As before, $u(s, \mathfrak{z})$ has two poles and a branching point. The long-time behavior is determined by the singularity with the largest real part, which is either the pole at $s = \frac{\alpha^2 v_0^2}{16D_h^3} - \frac{v_\parallel^2}{4D_h}$, when $\frac{\alpha^2 v_0^2}{16D_h^3} - \frac{v_\parallel^2}{4D_h} > 0$, or the pole at $s = 0$ otherwise. Let us consider the former possibility first, which occurs when $|v_\parallel| < \frac{\alpha v_0}{2D_h}$.

Upon evaluating the contribution of the dominant pole to the inverse Laplace transform and changing from u to h, we obtain that

$$h(t, x)_{\text{bulge}} = \left(1 + \frac{\alpha^2}{8D_h^2} - \frac{v_\parallel^2}{2v_0^2}\right)v_0 t - \frac{\alpha}{2D_h}|x - v_\parallel t| - \frac{v_\parallel}{v_0}(x - v_\parallel t). \tag{S55}$$

Compared to a non-translating bulge for $f^* = \frac{1}{2}$, a moving bulge exhibits a slower increase in height and an asymmetry between the leading and the trailing slopes. The leading slope is steeper while the trailing slope is shallower.

As expected, the peak of the bulge, which corresponds to the location of the in-flow boundary, moves with $v_\parallel$ relative to the x-axis. The spatial extent of the bulge is given by the locations where the sides of the bulge intersect the unperturbed flat front. The velocities of these bulge ends (or bulge feet) can be easily determined from Eq. (S55) by setting h to $v_0 t$. The results are

$$\begin{aligned} v_+ &= \frac{v_\parallel}{2} + \frac{\alpha v_0}{4D_h}, \\ v_- &= \frac{v_\parallel}{2} - \frac{\alpha v_0}{4D_h}, \end{aligned} \tag{S56}$$

where $+$ refers to the end on the right of the bulge and $-$ to the end on the left of the bulge.

Note that one of these velocities as well as the slope of the trailing side of the bulge approach 0 as the $|v_\parallel|$ increases towards $\frac{\alpha v_0}{2D_h}$. This limit corresponds to the exclusion of the less chiral strain. Indeed, the in-flow boundary advances with velocity $v_\parallel$ and a sharp slope at the leading edge. The shape of the leading edge does not change with time because the velocity of the corresponding bulge end also equals $v_\parallel$. The velocity of the trailing end, on the other hand, is 0, so it remains stationary. As a result, the more chiral strain continually gains more and more territory in the direction of its chirality. Consistent with this picture, Eq. (S55) also predicts that the slope of the trailing edge is 0 and the height of the bulge does not increase with time.

Once $|v_\parallel| > \frac{\alpha v_0}{2D_h}$, the pole at $s = 0$ becomes the dominant singularity, and the asymptotic solution for h changes to

$$h(t, x) = v_0 t + \frac{2D_h}{v_0}\ln\left(1 + \frac{1}{\frac{2D_h v_\parallel}{\alpha v_0} - 1}\left[\theta(x - v_\parallel t)e^{-\frac{v_\parallel}{D_h}(x - v_\parallel t)} + \theta(-x + v_\parallel t)\right]\right), \tag{S57}$$

where we assumed that $v_{\|} > 0$ to avoid cumbersome notation. The corresponding result for negative $v_{\|}$ can obtained by applying a mirror symmetry.

The behavior of the bulge described by Eq. (S57) is completely analogous to the dynamics in the limit of $v_{\|}$ approaching $\frac{\alpha v_0}{2D_h}$ from below that we just discussed. In particular, the leading edge advances with velocity $v_{\|}$ and has a time-invariant, in this case exponential, shape. The trailing edge has a zero slope, but a slightly higher height compared to the unperturbed flat front away from the in-flow boundary. Therefore, we conclude that, when $|v_{\|}| \geq \frac{\alpha v_0}{2D_h}$, the more chiral strain invades the less chiral strain, but the less chiral strain does not invade the more chiral strain.

S6 Natural selection due to the influence of front shape on the dynamics of $f(t,x)$

This section explains how bulge growth leads to natural selection via the mechanism illustrated in Fig.5 in the main text. The key idea is that the motion of an out-flow boundary changes once it comes in contact with the bulge. Here, we only consider $v_{\|} < \frac{\alpha v_0}{2D_h}$ because the mechanism of selection for $|v_{\|}| \geq \frac{\alpha v_0}{2D_h}$ has been explained in the preceeding section.

Boundary motion in the presence of bulges

For concreteness, let us assume that $v_{\|} > 0$ and that the bulge reaches the out-flow boundary from the left, as shown in Figs.5 and 6 in the main text. The analysis of the other cases is completely analogous.

The first thing to observe is that the contact of an out-flow boundary with a bulge is inevitable. An out-flow boundary located within a flat region of the front moves with velocity $v_{\|}$ specified by Eq. (S28). This velocity is opposite to the velocity of the left end of the bulge, v_-, and is smaller than the velocity of the right end of the bulge, v_+; see Eq. (S56). Thus, an out-flow boundary and one of the nearest bulges always meet.

The second thing to observe is that an out-flow boundary on the slope of a bulge catches up with the bulge end. To demonstrate this, we need to account for the effect of front tilt on boundary motion. Let us consider a region, such as the slope of the bulge, where $\frac{\partial h}{\partial x} = \varphi = \text{const}$. Equation (S24) then takes the following form:

$$\frac{\partial f}{\partial t} = D_f \frac{\partial^2 f}{\partial x^2} + \beta(f^* - f)\frac{\partial f}{\partial x} + v_0 \varphi \frac{\partial f}{\partial x}. \tag{S58}$$

Thus, the coupling between h and f leads to an effective advection with velocity $-v_0\varphi$, which modifies our expression for the boundary velocity in Eq. (S28) as follows

$$v_b = v_{\|} - v_0 \varphi, \tag{S59}$$

where we used v_b to denote the boundary velocity within a titled region of the front and $v_{\|}$ to denote the boundary velocity within a flat region of the front. Upon evaluating φ from Eq. (S55), we find that

$$v_b = 2v_{\|} + \frac{\alpha v_0}{2D_h} > v_+, \tag{S60}$$

i.e. an out-flow boundary on the slope of the bulge moves faster than the bulge end.

From the two observations above, we conclude that an out-flow boundary must localize at the end of the bulge. Indeed, the boundary can neither escape in the flat region ahead of the front nor fall behind on the slope of the bulge. While our asymptotic solution predicts a discontinous change in $\frac{\partial h}{\partial x}$ when the bulge and the flat front meet, it is clear that the slope of the front changes continuously. The inequality in Eq. (S60) ensures that there is a value of $\frac{\partial h}{\partial x}$ in this transition region such that Eq. (S59) is satisfied.

The origin of selection

After an out-flow boundary is trapped at the bulge end, it moves with velocity v_+ rather than $v_{\|}$. This leads to a change in the relative abundance of the two strains, i.e. to natural selection. The change in the strain fractions ceases only when the out-flow boundary is locked between the opposite slopes of the two nearest bulges. In the simulation shown in Fig.5 in the main text, the symmetry due to $f^* = 1/2$ ensures that the out-flow boundaries are exactly in the middle between the two bulges. Therefore, selection stops when the strains reach equal fractions. The dynamics for $f^* \neq 1/2$ are discussed next.

Negative frequency-dependent selection and the derivation of $\bar{f}_{\text{eq}}$

Since natural selection is mediated by the motion of out-flow boundaries, the steady state is reached when the out-flow boundaries are no longer moving relative to each other. This relative motion ceases when all out-flow boundaries are in contact with both of the nearest bulges as illustrated in Fig.6 in the main text. In this coexistence phase, the relative abundance of the strains can be quantified by $\bar{f}(t)$, the spatial average of $f(t, x)$.

The value of $\bar{f}_{\text{eq}}$ in equilibrium can be computed using the following geometrical argument; see Fig.6 in the main text. For small $\frac{\partial h}{\partial x}$, the height of the bulge equals the product of its slope and the horizontal extent of its side. This product must be the same for both sides of the bulge because the height of the bulge has a unique value. Therefore, the ratio of the side lengths is equal to the inverse ratio of the side slopes. Since the ratio of the slopes is the same for all bulges, so must be the ratio of the sizes of the domains occupied by each of the strains. The latter quantity is nothing but $\bar{f}_{\text{eq}}/(1 - \bar{f}_{\text{eq}})$, so we conclude that

$$\frac{\bar{f}_{\text{eq}}}{1 - \bar{f}_{\text{eq}}} = \frac{\frac{\partial h}{\partial x}\Big|_{\text{side occupied by strain 2}}}{\frac{\partial h}{\partial x}\Big|_{\text{side occupied by strain 1}}}. \tag{S61}$$

The value of $\bar{f}_{\text{eq}}$ can then be obtained by using Eq. (S55) to determine bulge slopes and Eq. (S28) to determine $v_{\|}$. The result reads

$$\bar{f}_{\text{eq}} = \frac{1}{2} + \frac{\beta D_h}{\alpha v_0}\left(f^* - \frac{1}{2}\right). \tag{S62}$$

For $|f^* - \frac{1}{2}| < \frac{\alpha v_0}{2 D_h \beta}$, Eq. (S62) predicts that $\bar{f}_{\text{eq}} \in (0, 1)$, i.e. both strains are present at steady state. When this inequality is violated, $|v_{\|}| \geq \frac{\alpha v_0}{2 D_h}$, and one of the strains goes extinct as we discussed above. This transition occurs at $f_c^* = \frac{1}{2} \pm \frac{\alpha v_0}{2 D_h \beta}$, which could lie both within and outside $[0, 1]$. In the former case, the competition of the non-chiral strain vs. a chiral strain ($f^* = 0$ or $f^* = 1$) always results in the extinction of the less chiral strain. In the latter case, a chiral and a non-chiral strains coexist.

To avoid possible confusion, we emphasize that, unlike the value of $\bar{f}$, the value of f^* is not constrained to lie between 0 and 1; see Eq. (S23). The values of f^* outside $[0, 1]$ correspond to strains that have different magnitudes of chirality, but are chiral in the same direction (i.e. $A^{(1)}$ and $A^{(2)}$ have the same sign). In particular, two strains with the same chirality correspond to $\alpha = 0$, $\beta = 0$, $f^* = \infty$, and $\beta f^* =$ finite.

S7 Off-lattice simulations

Here we describe the simulations that were performed without introducing a lattice of sub-populations and, therefore, without breaking the rotational invariance of the space. These simulations provide an important confirmation of our theoretical results because lattice effects are known to create many artifacts especially in the presence of chirality [20].

In off-lattice simulations, each cell i has position (x_i, y_i) which are continuous variables without an underlying lattice. Cell growth and movement depends on the arrangement of other cells through the effective population density, $C(x, y)$ and its gradient $\nabla C(x, y)$. The effective population density sensed by cell i is given by

$$C(x_i, y_i) = \sum_j \frac{1}{N} e^{-(x_i - x_j)^2 - (y_i - y_j)^2}, \tag{S63}$$

where the sum is over the entire population of cells, and N is the carrying capacity which normalizes C to be order 1 in the bulk of the population. Note that the Gaussian kernel is rotationally and translationally symmetric. The gradient of the population density is computed from its definition and is given by

$$\nabla C(x, y) = -2 \sum_j \frac{1}{N} e^{-(x - x_j)^2 - (y - y_j)^2} \left[(x - x_j)\hat{\mathsf{x}} + (y - y_j)\hat{\mathsf{y}}\right]. \tag{S64}$$

A cell i at (x_i, y_i) gives birth to a daughter cell at the same position with probability $g_i(1 - C(x_i, y_i))$, where g_i is the growth rate of the cell i. If the region is overcrowded, the growth probability is negative and the cell dies with the negative of this growth probability.

A cell at (x_i, y_i) jumps with a probability proportional to the population density $C(x_i, y_i)$. This cooperative migration ensures that the wave is pushed. The jump size is fixed to a value μ. Since cell motion is chiral, the direction of the jump is biased by the gradient in the concentration. The angle of the jump of cell i with chirality A_i is termed θ_i. This angle is chosen from a distribution with mean ϕ_i such that

$$\tan \phi_i = -\frac{\nabla C(x_i, y_i).\hat{\mathsf{x}}}{\nabla C(x_i, y_i).\hat{\mathsf{y}}} \tag{S65}$$

where we ensure that ϕ_i is in the correct quadrant accounting for the sign of the chirality. The orientation of ϕ relative to the gradient was 90° counterclockwise for positive chirality and 90° clockwise for negative chirality. Although the mean direction of the jump could be at an arbitrary angle with respect to the gradient in the most the most general model, we restrict ourselves to this case which was sufficient to explore the effects of chirality. The angle of the jump, θ_i is then chosen from the wrapped normal distribution

$$P(\theta_i) = \sqrt{\frac{|\nabla C(x_i, y_i)||A_i|}{\pi^2}} \sum_{k=-\infty}^{k=\infty} \mathsf{exp}\left[-\frac{(\theta_i - \phi_i + 2\pi k)^2}{\pi} |\nabla C(x_i, y_i)||A_i|\right], \tag{S66}$$

where θ_i lies in $[0, 2\pi)$. The variance of the normal distribution is inversely proportional to magnitude of the chirality of the cell and the population gradient. This ensures that the angle of the jump is sharply peaked

if the cell is very chiral and the gradient is strong. When either the gradient or the chirality is weak, the distribution tends to a uniform distribution which leads to unbiased diffusion. To suppress migration into crowded areas, a jump to position (x_d, y_d) is rejected with probability $(1 - C(x_d, y_d))$. This freezes dynamics in the colony bulk like in the experiments.

In simulations, the cells grow in a semi-infinite box extending in the $+y$ direction, with finite width W and periodic boundary conditions in the x direction. We use synchronous updates: first the effective population density and its gradient are computed for every cell, then the cells are allowed to grow or die, and finally the migration step is performed. To speed up simulations, we only simulate cells near the front, as the dynamics in the colony bulk are frozen and activity in the bulk has no effect on the front dynamics.

S1 Fig. Quantitative test of Eq. (5), the relationship between $\bar{f}_{\rm eq}$ and f^*. **(A)** shows the shape (shape preserving spline) of an asymmetric bulge from a simulation with $f^* = 0.75$. The red lines are the best fit of the two slopes of the bulge. From these slopes, we obtained $\frac{\alpha}{D_h} = 0.080$ and $\frac{\beta}{v_0} = 0.068$ using Eq. (S55); the values of these parameters are averages over all runs with $f^* \neq 0.5$. **(B)** shows $\bar{f}_{\rm eq}$ from simulations (dots) and the theoretical prediction (line) from Eq. (5) in the main text and the estimated values of the parameters. The predicted slope equals 0.856 and is quite close to 0.82 ± 0.02, which is the slope obtained by ordinary least squares regression (not shown). The root mean square deviation between the theory and the simulations is 0.01. Here, $m_0 = m_s = m_b = m_d = 0$, $g = 0.1$, $m_l + m_r = 0.01$ for both strains. Simulations started from two separate domains on a lattice of 1200x14000 sites with $N = 800$. We ensured that the simulations reached steady state by starting runs from an initial fractions of 0.25, 0.5 and 0.75. Error bars (s.e.m) were estimated from a set of 18 runs with 6 starting from each initial fraction.

S2 Fig. The size of the coexistence region depends on model parameters. This figure is the same as Fig. 7B, but for different model parameters. In comparison with the figure in the main text, the coexistence region is smaller, and the slope of $\bar{f}_{\rm eq}$ is steeper. The green line is the least squares fit to the simulation data (dots); the slope is 1.45, and $R^2 = 0.997$. The solid part of the line spans the data points with $\bar{f}_{\rm eq} \in (0, 1)$ that were used in the fit. The dashed part extends this dependence to the entire region of possible $\bar{f}_{\rm eq}$. The black dashed line marks the unit slope. Here, $m_0 = 0.01, m_s = m_b = m_d = 0$, $g = 0.1$, $m_l + m_r = 0.1$ for both strains. Simulations started from well mixed conditions on a lattice of width 300 sites with $N = 200$. The simulation time was chosen to ensure that the same steady state was approached starting from initial conditions that are both above and below $\bar{f}_{\rm eq}$.

S3 Fig. Genetic drift, but not chirality control the boundary width between identical strains. **(A)** shows that domain boundaries between strains with equal chirality become wider for larger N (weaker genetic drift); the dependence is approximately linear in agreement with Ref. [17]. **(B)** In contrast, chirality has no detectable effect on the boundary width. The rest of the panels show the spatial patterns used to reach these conclusions. The boundary width was computed as the local heterozygosity, $2\langle f(1-f)\rangle$, summed over the entire width of the simulation and averaged over $y \in (2000, 4000)$. Simulations were performed on a lattice of 1000x4000 sites with $m_0 = m_s = m_b = m_d = 0$, $g = 0.1$.

S4 Fig. Mixing transitions at different values of relative chirality. **(A), (B), (C)** show demixed phases for strong genetic drift and varying values of f^*. **(D), (E)** shows dissolution of a boundary and the establishment of the intermixed phase for weak genetic drift and two values of f^*. **(F)** shows the competition between a chiral and a non-chiral strain for weak genetic drift. The boundary is much wider than in (C), but no intermixed phase is established because the non-chiral strain is outcompeted. Here, $m_0 = m_s = m_b = m_d = 0$, $g = 0.1$, $m_l + m_r = 0.1$ for both strains. Simulations were carried out on a lattice of 600x3000 sites.

S5 Fig. Chirality differences, but not genetic drift control the time scale of selection. **(A)** shows that the fixation time of the chiral strain decreases with the magnitude of its chirality. Similarly, **(B)** demonstrates that stronger chirality results in shorter equilibration times for two strains with opposite chirality. **(C)**, and **(D)** show that genetic drift does not affect the time scale of selection. Here, $m_0 = m_s = m_b = m_d = 0$, $g = 0.1$, $m_l + m_r = 0.1$ for both strains. In (A) and (B), simulations were started from well-mixed initial conditions with $N = 400$ on a lattice of 500x6000 and 1000x1200 sites respectively. Simulations started from two demixed domains with $m_l^{(1)} = 0.09$, $m_r^{(1)} = 0.01$, $m_l^{(2)} = 0.05$, $m_r^{(2)} = 0.05$, on a lattice of 500x7500 sites in (C), and $m_l^{(1)} = 0.09$, $m_r^{(1)} = 0.01$, $m_l^{(2)} = 0.01$, $m_r^{(2)} = 0.09$ on a lattice of 500x3000 sites in (D).

S6 Fig. Effects of chirality on the expansion velocity. In panel **(A)**, we demonstrate that a change in chirality does not produce a change in the expansion velocity of a strain grown in isolation when $m_l + m_r$

is kept fixed. **(B)** shows that the expansion velocity increases, but only slightly, when two strains with opposite handedness expand together. Here, $m_0 = m_s = m_b = m_d = 0$, $g = 0.1$, $m_l + m_r = 0.1$ for both strains. Simulations started from well-mixed conditions on a lattice of 200x1000 sites with $N = 200$. Error bars (s.e.m) were estimated from 4 identical runs and velocity is measured in units of lattice spacing.

S7 Fig. Chirality affects competition in off-lattice simulations. (A) shows the emergence of a bulge between two oppositely chiral strains. **(B)** shows the stabilizing selection between two oppositely chiral strains similar to Fig. 3C in the main text. The species fractions were averaged over 6 runs. Parameters were $N = 6$, $A^{(1)} = 10$, $A^{(2)} = -10$, $g^{(1)} = g^{(2)} = 0.2$ for both figures, and $W = 200$, $\mu = 1.0$ in (A) and $W = 500$, $\mu = 0.5$ in (B). Distances were rescaled by a factor of 100, similar to simulations on the lattice in the text.

References

[1] Van Saarloos W. Front propagation into unstable states. Physics Reports. 2003;386:29–222.

[2] Murray JD. Mathematical Biology. Springer; 2003.

[3] Birzu G, Hallatschek O, Korolev KS. Fluctuations uncover a distinct class of traveling waves. Proceedings on the National Academy of Sciences, USA. 2018;Early Edition.

[4] Balasuriya S, Gottwald GA. Wavespeed in reaction–diffusion systems, with applications to chemotaxis and population pressure. Journal of mathematical biology. 2010;61(3):377–399.

[5] Paquette GC, Chen LY, Goldenfeld N, Oono Y. Structural stability and renormalization group for propagating fronts. Physical review letters. 1994;72(1):76.

[6] Birzu G, Matin S, Hallatschek O, Korolev KS. Density-dependent dispersal can preserve genetic diversity during range expansions. 2018;.

[7] Kardar M, Parisi G, Zhang YC. Dynamic scaling of growing interfaces. Physical Review Letters. 1986;56(9):889.

[8] Sasamoto T, Spohn H. One-dimensional Kardar-Parisi-Zhang equation: an exact solution and its universality. Physical review letters. 2010;104(23):230602.

[9] Drossel B, Kardar M. Phase Ordering and Roughening on Growing Films. Phys Rev Lett. 2000;85:614–617.

[10] Hallatschek O, Nelson DR. Gene surfing in expanding populations. Theoretical Population Biology. 2008;73:158–170.

[11] Bateman H. Some recent researches on the motion of fluids. Monthly Weather Review. 1915;43(4):163–170.

[12] Burgers JM. A mathematical model illustrating the theory of turbulence. Advances in applied mechanics. 1948;1:171–199.

[13] Sachdev PL. Nonlinear diffusive waves. Cambridge University Press; 1987.

[14] Whitham GB. Linear and nonlinear waves. vol. 42. John Wiley & Sons; 2011.

[15] Korolev KS, Avlund M, Hallatschek O, Nelson DR. Genetic demixing and evolution in linear stepping stone models. Reviews of modern physics. 2010;82(2):1691.

[16] Korolev KS, Xavier JB, Nelson DR, Foster KR. A quantitative test of population genetics using spatiogenetic patterns in bacterial colonies. The American Naturalist. 2011;178:538–552.

[17] Hallatschek O, Korolev KS. Fisher waves in the strong noise limit. Physical Review Letters. 2009;103(10):108103.

[18] Hallatschek O, Hersen P, Ramanathan S, Nelson DR. Genetic drift at expanding frontiers promotes gene segregation. Proceedings of the National Academy of Sciences. 2007;104(50):19926–19930.

[19] Gradshteyn I, Ryzhik I. Table of Integrals, Series, and Products. Academic Press, New York; 1980.

[20] Tucker LJ. A new computational approach to simulate pattern formation in Paenibacillus dendritiformis bacterial colonies. University of California, San Diego; 2010.
