# Supplementary figures and images for: Chirality provides a direct fitness advantage and facilitates intermixing in cellular aggregates
Source: PLoS Comput Biol. 2018 Dec 27;14(12):e1006645. doi: 10.1371/journal.pcbi.1006645 (PMC6307711; doi:10.1371/journal.pcbi.1006645)

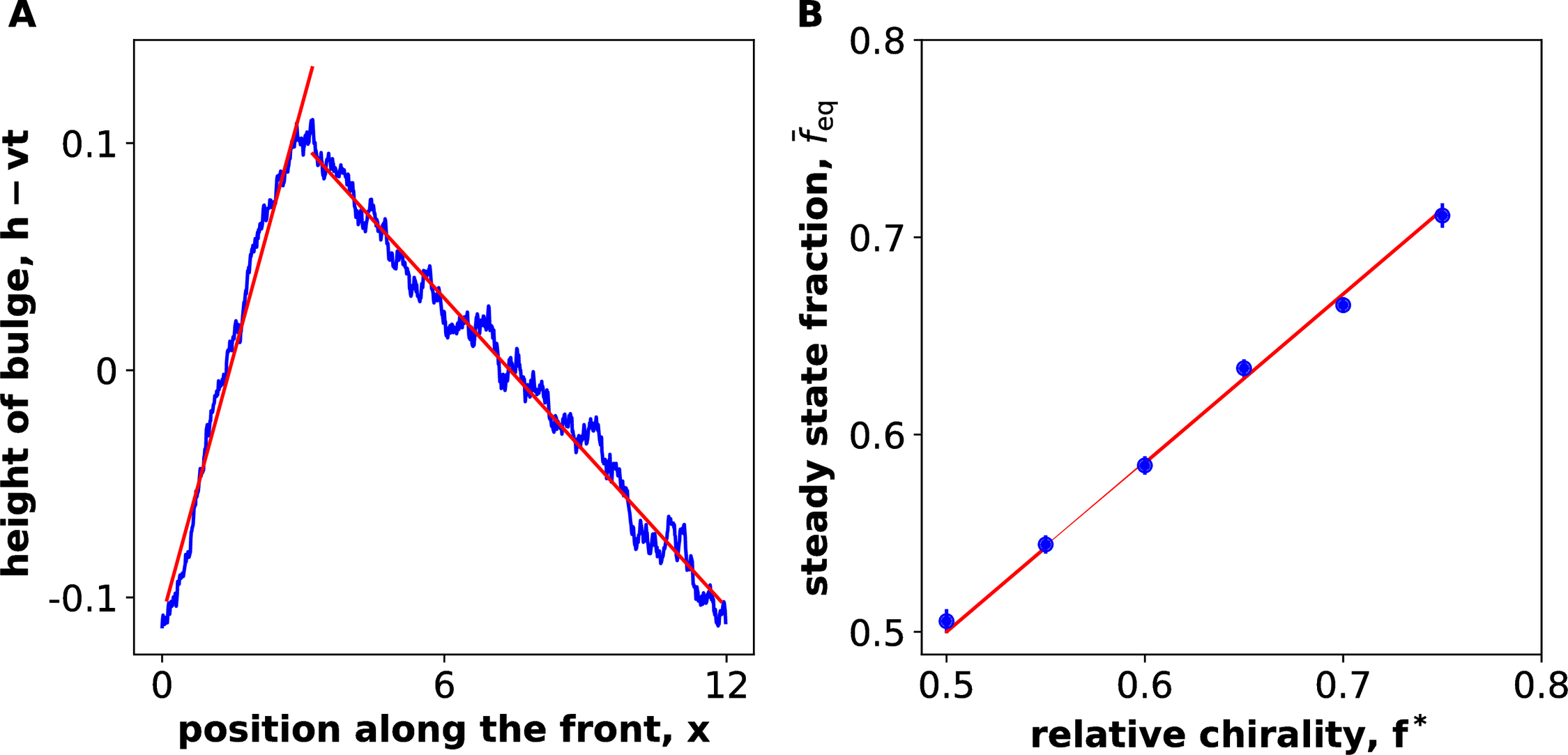

Supplement: S1 Fig — (A) shows the shape (shape preserving spline) of an asymmetric bulge from a simulation with f* = 0.75. The red lines are the best fit of the two slopes of the bulge. From these slopes, we obtained αDh=0.080 and βv0=0.068 using Eq. (S55); the values of these parameters are averages over all runs with f* ≠ 0.5. (B) shows f‾eq from simulations (dots) and the theoretical prediction (line) from Eq (5) in the main text and the estimated values of the parameters. The predicted slope equals 0.856 and is quite close to 0.82 ± 0.02, which is the slope obtained by ordinary least squares regression (not shown). The root mean square deviation between the theory and the simulations is 0.01. Here, m0 = ms = mb = md = 0, g = 0.1, ml + mr = 0.01 for both strains. Simulations started from two separate domains on a lattice of 1200x14000 sites with N = 800. We ensured that the simulations reached steady state by starting runs from an initial fractions of 0.25, 0.5 and 0.75. Error bars (s.e.m) were estimated from a set of 18 runs with 6 starting from each initial fraction. (TIF) [file pcbi.1006645.s002.tif]

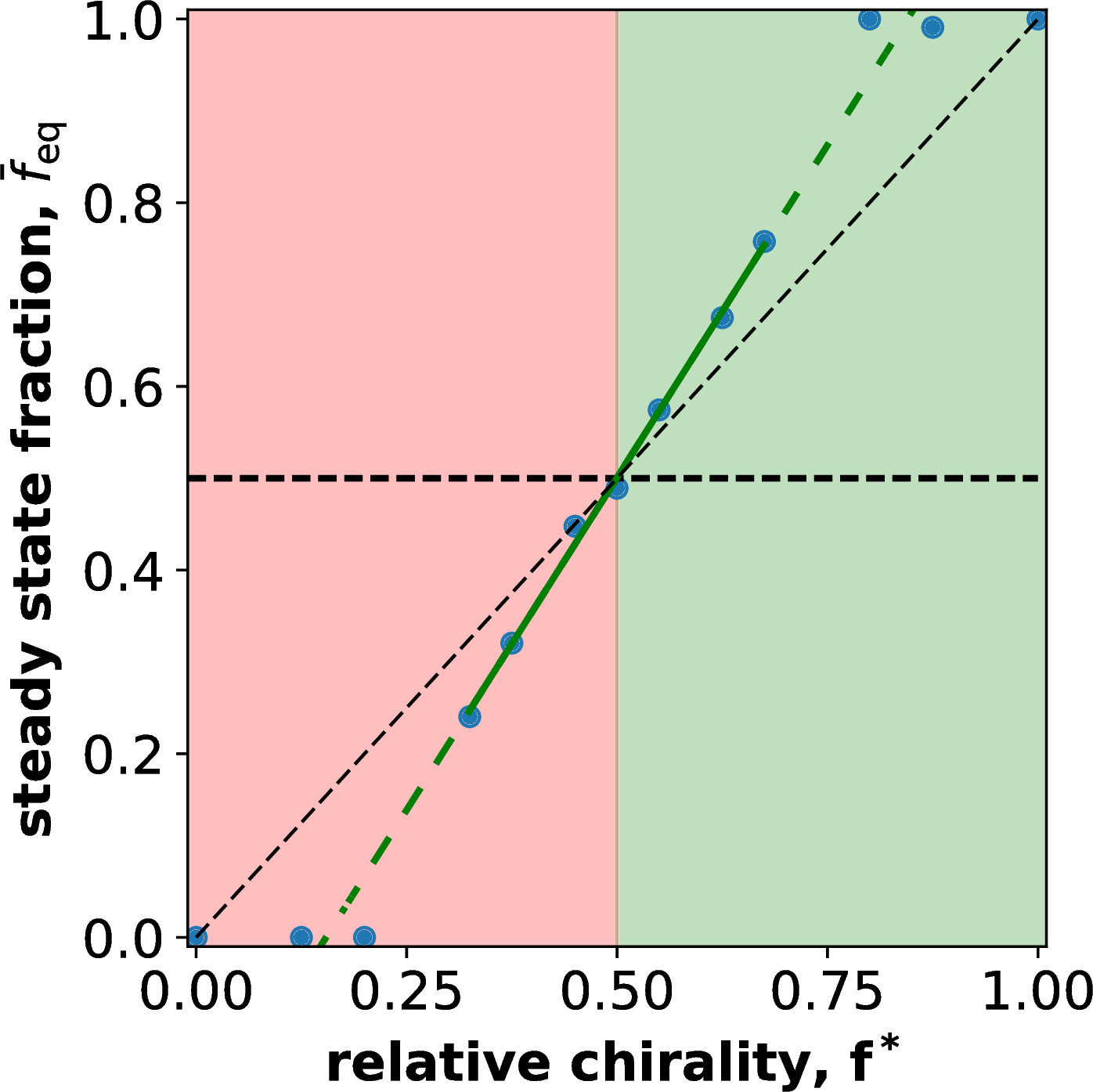

Supplement: S2 Fig — This figure is the same as Fig 7B, but for different model parameters. In comparison with the figure in the main text, the coexistence region is smaller, and the slope of f‾eq is steeper. The green line is the least squares fit to the simulation data (dots); the slope is 1.45, and R2 = 0.997. The solid part of the line spans the data points with f‾eq∈(0,1) that were used in the fit. The dashed part extends this dependence to the entire region of possible f‾eq. The black dashed line marks the unit slope. Here, m0 = 0.01, ms = mb = md = 0, g = 0.1, ml + mr = 0.1 for both strains. Simulations started from well mixed conditions on a lattice of width 300 sites with N = 200. The simulation time was chosen to ensure that the same steady state was approached starting from initial conditions that are both above and below f‾eq. (TIF) [file pcbi.1006645.s003.tif]

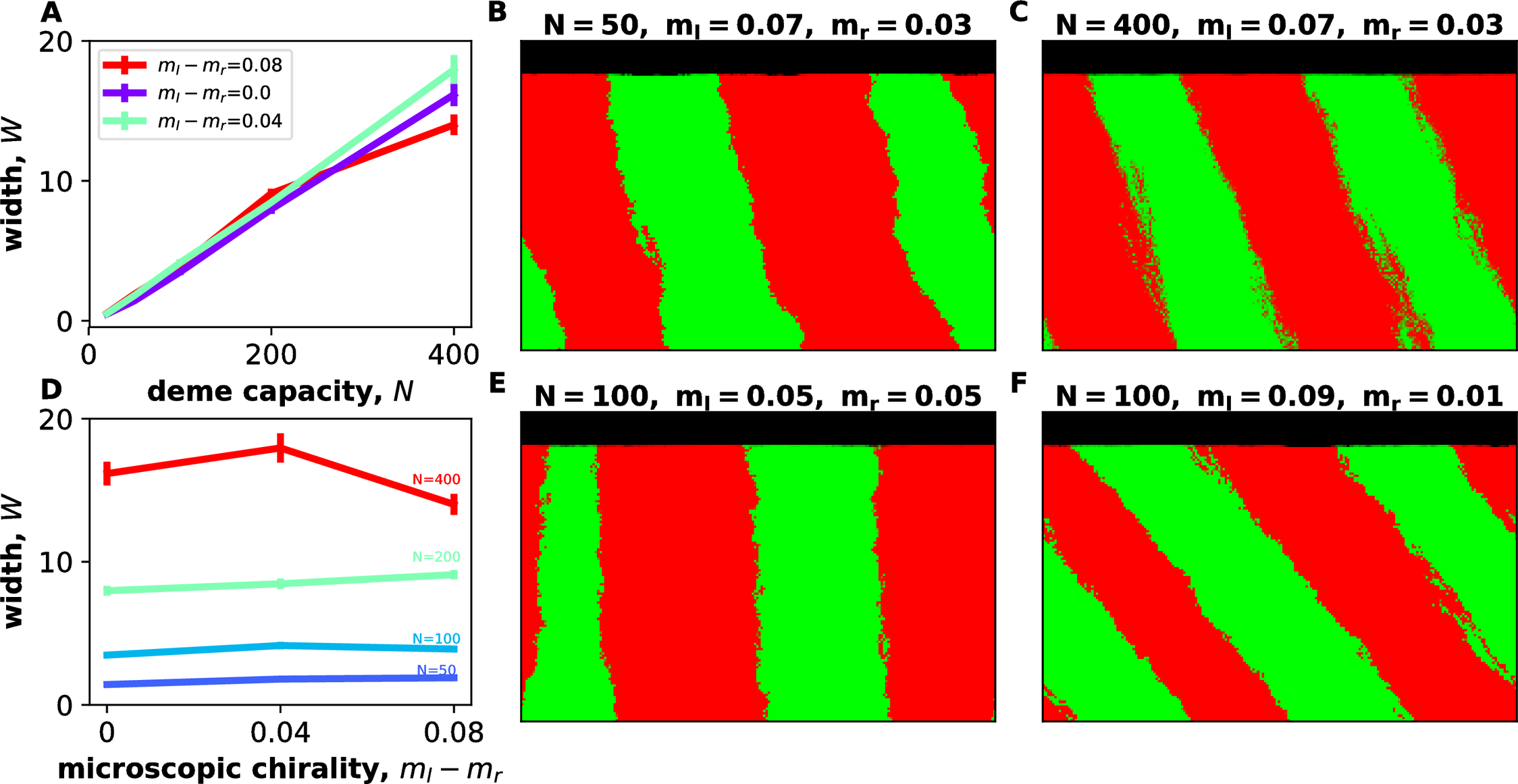

Supplement: S3 Fig — (A) shows that domain boundaries between strains with equal chirality become wider for larger N (weaker genetic drift); the dependence is approximately linear in agreement with Ref. [63]. (B) In contrast, chirality has no detectable effect on the boundary width. The rest of the panels show the spatial patterns used to reach these conclusions. The boundary width was computed as the local heterozygosity, 2〈f(1 − f)〉, summed over the entire width of the simulation and averaged over y ∈ (2000, 4000). Simulations were performed on a lattice of 1000x4000 sites with m0 = ms = mb = md = 0, g = 0.1. (TIF) [file pcbi.1006645.s004.tif]

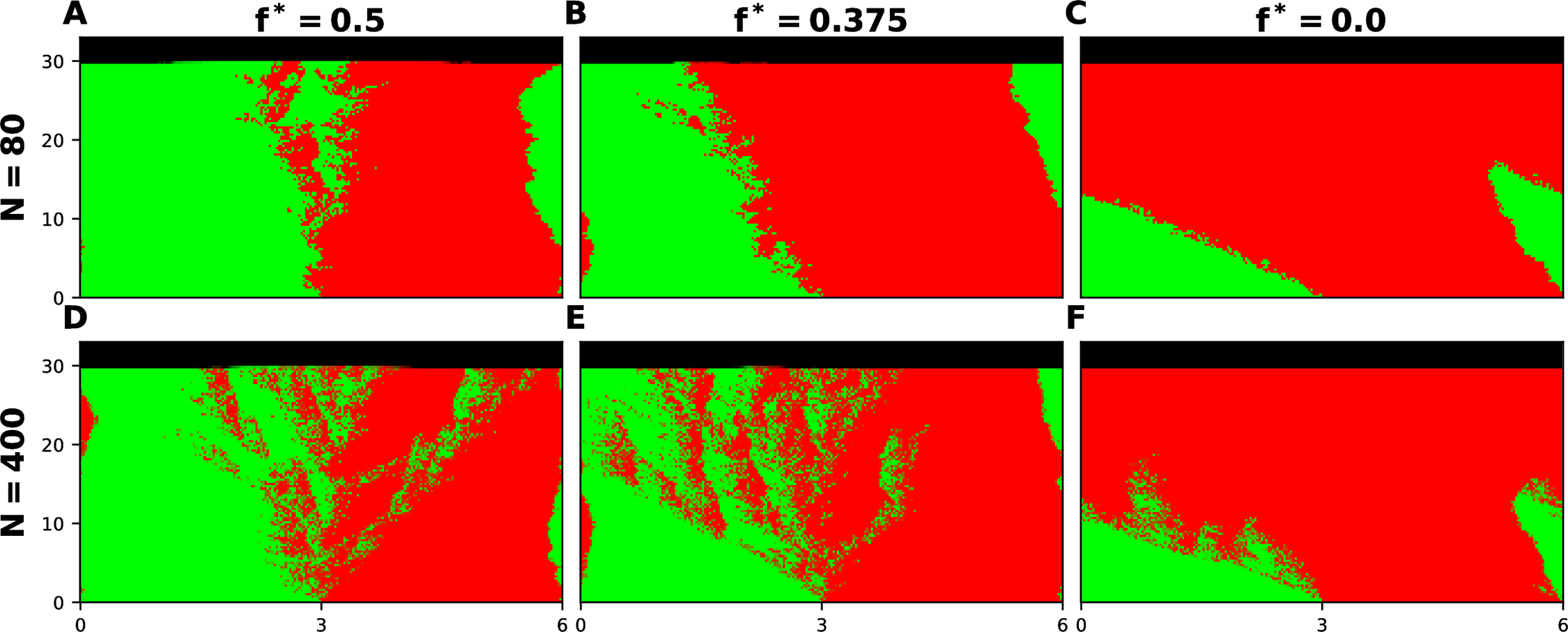

Supplement: S4 Fig — (A), (B), (C) show demixed phases for strong genetic drift and varying values of f*. (D), (E) shows dissolution of a boundary and the establishment of the intermixed phase for weak genetic drift and two values of f*. (F) shows the competition between a chiral and a non-chiral strain for weak genetic drift. The boundary is much wider than in (C), but no intermixed phase is established because the non-chiral strain is outcompeted. Here, m0 = ms = mb = md = 0, g = 0.1, ml + mr = 0.1 for both strains. Simulations were carried out on a lattice of 600x3000 sites. (TIF) [file pcbi.1006645.s005.tif]

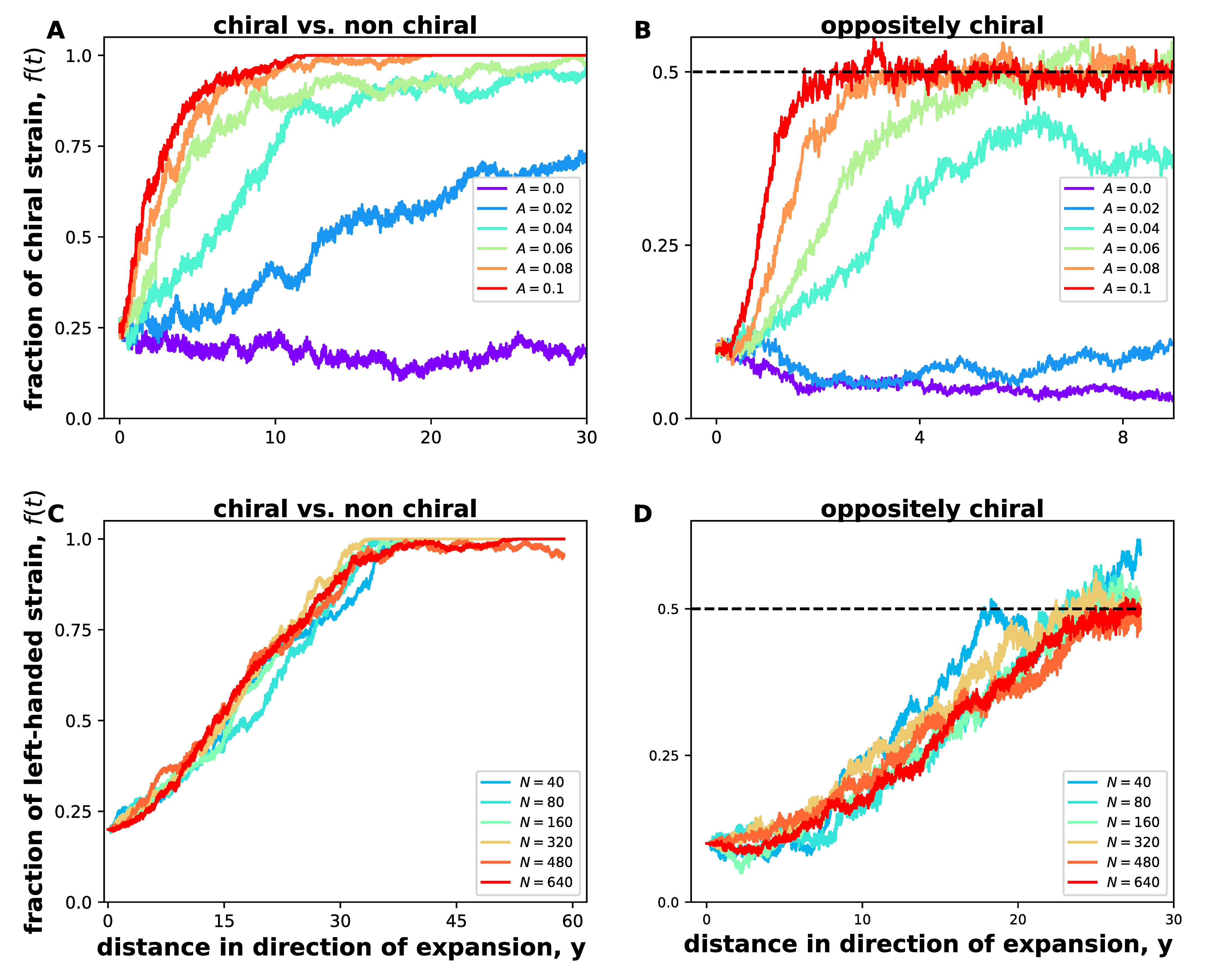

Supplement: S5 Fig — (A) shows that the fixation time of the chiral strain decreases with the magnitude of its chirality. Similarly, (B) demonstrates that stronger chirality results in shorter equilibration times for two strains with opposite chirality. (C), and (D) show that genetic drift does not affect the time scale of selection. Here, m0 = ms = mb = md = 0, g = 0.1, ml + mr = 0.1 for both strains. In (A) and (B), simulations were started from well-mixed initial conditions with N = 400 on a lattice of 500x6000 and 1000x1200 sites respectively. Simulations started from two demixed domains with ml(1)=0.09, mr(1)=0.01, ml(2)=0.05, mr(2)=0.05, on a lattice of 500x7500 sites in (C), and ml(1)=0.09, mr(1)=0.01, ml(2)=0.01, mr(2)=0.09 on a lattice of 500x3000 sites in (D). (TIF) [file pcbi.1006645.s006.tif]

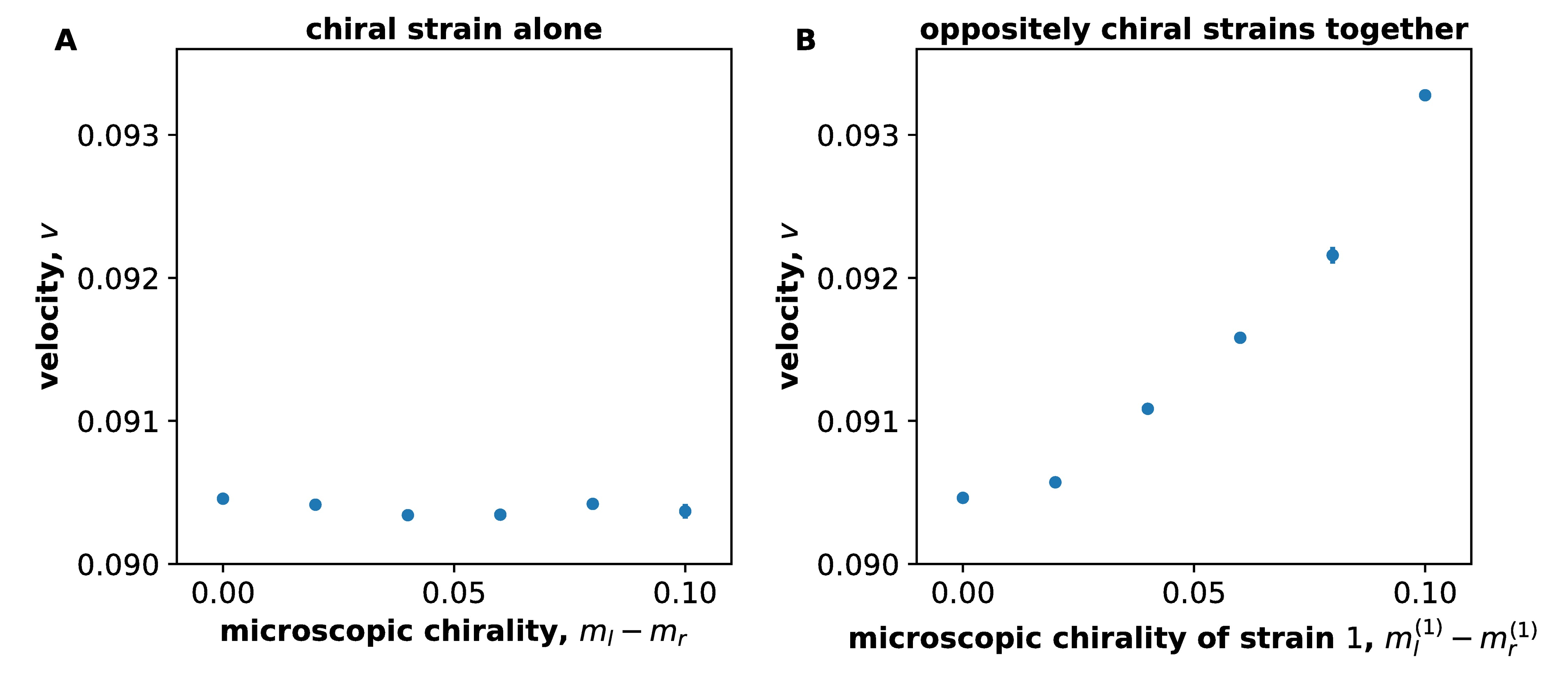

Supplement: S6 Fig — In panel (A), we demonstrate that a change in chirality does not produce a change in the expansion velocity of a strain grown in isolation when ml + mr is kept fixed. (B) shows that the expansion velocity increases, but only slightly, when two strains with opposite handedness expand together. Here, m0 = ms = mb = md = 0, g = 0.1, ml + mr = 0.1 for both strains. Simulations started from well-mixed conditions on a lattice of 200x1000 sites with N = 200. Error bars (s.e.m) were estimated from 4 identical runs and velocity is measured in units of lattice spacing. (TIF) [file pcbi.1006645.s007.tif]

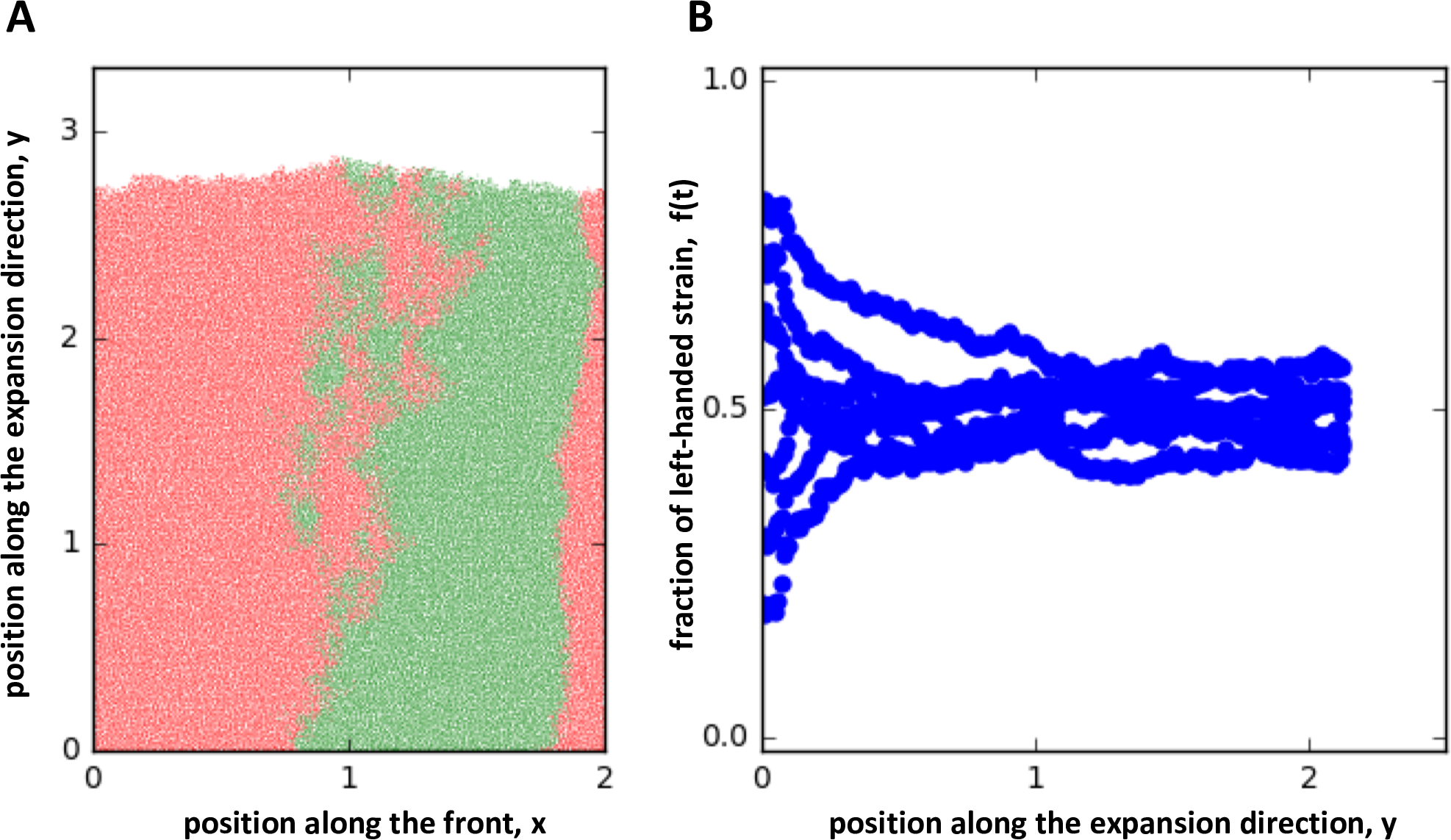

Supplement: S7 Fig — (A) shows the emergence of a bulge between two oppositely chiral strains. (B) shows the stabilizing selection between two oppositely chiral strains similar to Fig 3C in the main text. The species fractions were averaged over 6 runs. Parameters were N = 6, A(1) = 10, A(2) = −10, g(1) = g(2) = 0.2 for both figures, and W = 200, μ = 1.0 in (A) and W = 500, μ = 0.5 in (B). Distances were rescaled by a factor of 100, similar to simulations on the lattice in the text. (TIF) [file pcbi.1006645.s008.tif]
